# Migration of Type III Secretion System Transcriptional Regulators Links Gene Expression to Secretion

**DOI:** 10.1128/mBio.01096-18

**Published:** 2018-07-31

**Authors:** Spyridoula N. Charova, Anastasia D. Gazi, Efstratios Mylonas, Charalambos Pozidis, Blanca Sabarit, Dimitrios Anagnostou, Konstantina Psatha, Michalis Aivaliotis, Carmen R. Beuzon, Nickolas J. Panopoulos, Michael Kokkinidis

**Affiliations:** a Institute of Molecular Biology and Biotechnology, Foundation for Research and Technology–Hellas (IMBB-FORTH), Heraklion, Crete, Greece; b Department of Biology, University of Crete, Heraklion, Crete, Greece; c Ultrastructural BioImaging Unit (UTechs UBi), Department of Cellular Biology and Infection, Center for Innovation and Technological Research (CITECH), Institut Pasteur, Paris, France; d Instituto de Hortofruticultura Subtropical y Mediterranea La Mayora, Universidad de Málaga-Consejo Superior de Investigaciones Científicas (IHSM-UMA-CSIC), Departamento de Biología Celular, Genética y Fisiología, Málaga, Spain; e Laboratory of Biological Chemistry, School of Medicine, Faculty of Health Sciences, Aristotle University of Thessaloniki, Greece; Mass General Hospital

**Keywords:** Erwinia amylovora, Pseudomonas syringae pv. phaseolicola, type III secretion system (T3SS), gatekeeper complex

## Abstract

Many plant-pathogenic bacteria of considerable economic importance rely on type III secretion systems (T3SSs) of the Hrc-Hrp 1 family to subvert their plant hosts. T3SS gene expression is regulated through the HrpG and HrpV proteins, while secretion is controlled by the gatekeeper HrpJ. A link between the two mechanisms was so far unknown. Here, we show that a mechanistic coupling exists between the expression and secretion cascades through the direct binding of the HrpG/HrpV heterodimer, acting as a T3SS chaperone, to HrpJ. The ternary complex is docked to the cytoplasmic side of the inner bacterial membrane and orchestrates intermediate substrate secretion, without affecting early substrate secretion. The anchoring of the ternary complex to the membranes potentially keeps HrpG/HrpV away from DNA. In their multiple roles as transcriptional regulators and gatekeeper chaperones, HrpV/HrpG provide along with HrpJ potentially attractive targets for antibacterial strategies.

## IMPORTANCE

On the basis of scientific/economic importance, Pseudomonas syringae and Erwinia amylovora are considered among the top 10 plant-pathogenic bacteria in molecular plant pathology. Both employ type III secretion systems (T3SSs) of the Hrc-Hrp 1 family to subvert their plant hosts. For Hrc-Hrp 1, no functional link was known between the key processes of T3SS gene expression and secretion. Here, we show that a mechanistic coupling exists between expression and secretion cascades, through formation of a ternary complex involving the T3SS proteins HrpG, HrpV, and HrpJ. Our results highlight the functional and structural properties of a hitherto-unknown complex which orchestrates intermediate T3SS substrate secretion and may lead to better pathogen control through novel targets for antibacterial strategies.

## INTRODUCTION

Plant-pathogenic bacteria that cause major economic losses for the food and agriculture industry worldwide employ a type III secretion system (T3SS) from the Hrc-Hrp 1 family (hypersensitive response and pathogenicity [*hrp*] genes conserved) to infect and colonize their plant hosts (1). Among them are pathovars of Pseudomonas syringae, which collectively infect over 40 important crops worldwide (e.g., tomato, corn, pea, wheat, rice, soybean, etc.), and Erwinia amylovora, causing extensive crop losses due to fire blight in plants of the *Rosaceae* family (e.g., pear and apple). Both pathogens have been classified among the top 10 plant-pathogenic bacteria on the basis of their scientific and economic importance (2).

*P. syringae* employs the best-studied plant-pathogenic T3SS (3). This multiprotein assembly consists of a basal structure residing in the cell envelope and an extracellularly protruding pilus. It also comprises transcriptional regulators, specialized chaperones that escort certain secretion substrates, a variety of secretion substrates which include T3SS helpers (e.g., harpins/translocators which render the host cell permeable to translocated substrates), and T3SS effectors, the virulent weaponry of the pathogen (4).

At the level of T3SS gene expression, the Hrc-Hrp 1 operons bear a promoter consensus sequence known as the *hrp*-box, which is recognized by HrpL (5), an alternative σ factor. Two T3SS-specific enhancer-binding proteins (EBPs), HrpR and HrpS, have been found in *P. syringae* pathovars to associate into active heterohexamers to induce transcription of *hrpL* (6), while in *E. amylovora* this function is performed by homohexameric HrpS (7).

An additional protein, HrpV, serves in *P. syringae* as a negative regulator of the T3SS, binding and altering the oligomerization state of HrpS and suppressing productive interactions between the HrpR/HrpS heterohexamer and the closed promoter complex (6, 8). The HrpG protein has been shown to partially attenuate the negative regulation exerted by HrpV on the HrpR/HrpS complex, through direct binding which inhibits HrpV-HrpS interactions (6, 9). This double-negative regulatory loop imposed by the HrpV and HrpG proteins on the system is responsible for establishing a state of bistability on T3SS gene expression, with the bacteria differentiating stochastically into T3SS-expressing and nonexpressing populations within a homogeneous environment (10). We have recently reported the formation of a HrpG/HrpV complex in *E. amylovora*, which strongly suggests a general regulatory pathway controlling the transcriptional activation in the Hrc-Hrp 1 family (11).

At the level of T3SS secretion in animal pathogens, proteins from the gatekeeper family play a central role, along with heterodimeric class I T3SS chaperones, with which they frequently associate. They serve as T3SS plugs, preventing premature secretion of effectors and yet permitting the exit of helpers, until a switching event takes place, possibly triggered by host-derived stimuli (12, 13). The gatekeeper proteins from plant and animal T3SSs exhibit several analogies and are organized in one or two separate polypeptide chains (14). In the phytopathogenic Hrc-Hrp 1 system, the gatekeeper HrpJ is a secreted and translocated substrate required for the secretion of helpers, the subsequent translocation of effectors, and the elicitation of the hypersensitive response (HR) (15, 16). Following its translocation, HrpJ also plays a role inside the plant, contributing to the suppression of host immunity (16).

Until now, no direct connection between the processes of T3SS gene expression regulation and T3SS protein secretion by employing the same protein components was established. In this work, we show that in Pseudomonas syringae pv. phaseolicola and *E. amylovora* the transcriptional regulators HrpG and HrpV assume, after migration toward the inner bacterial membrane, the role of a T3SS chaperone and associate with the gatekeeper HrpJ, thereby forming a membrane-docked ternary complex. This promotes secretion of intermediate T3SS substrates, e.g., the harpin HrpZ1, without affecting early substrate secretion (12). Thus, formation of the ternary complex fine-tunes the regulation of secretion and expression mechanisms. Biochemical and structural characterization of the HrpG/HrpV/HrpJ complex reveals conserved gatekeeper interaction patterns across various T3SSs and a key role of HrpG in docking the complex to bacterial membranes. A new and potentially broadly applicable concept for the coregulation of T3SS transcription and secretion by component migration emerges from these studies.

## RESULTS

### Gatekeeper chaperone genes are located upstream of the T3SS secretin genes in a wide range of T3SS pathogens.

Sequence comparisons classify HrpJ from *P. syringae* pv. phaseolicola and *E. amylovora* as members of the YopN/TyeA family of T3SS proteins (15). These proteins (e.g., MxiC, SsaL, SepL, and InvE), including HrpJ, have been found to possess two domains with extensive amino acid sequence homologies (see Fig. S1 in the supplemental material) and usually associate with an atypical, heterodimeric class I T3SS chaperone (17), in contrast to the typical class I chaperones, which are homodimeric escorts of T3SS effectors (18). Structural information is available for YopN, a counterpart of HrpJ from the T3SS of Yersinia pestis, which associates with its cognate chaperone, the SycN/YscB heterodimer, via two β-motifs (Fig. S1A), with each β-motif interacting with a different subunit of the chaperone (17, 18). The HrpJ sequence from *P. syringae* pv. phaseolicola and *E. amylovora* was examined for the presence of comparable β-motifs, and significant homologies were detected with the second β-motif (Fig. S1A), which is conserved in several members of the YopN/TyeA family. This probably reflects a similar pattern of gatekeeper-chaperone interactions extending across various species and T3SS families.

10.1128/mBio.01096-18.2 FIG S1  Multiple sequence alignment of the T3SS gatekeeper domains. (A) Multiple sequence alignment of the N-terminal domain of the gatekeeper proteins highlighting chaperone binding β-motifs. Multiple sequence alignment between full-length gatekeeper sequences was performed using COBALT. Only part of the alignment is shown, comprising the N-terminal YopN-like domains of the gatekeeper proteins. Aligned sequence stretches with no gaps are colored in blue or red. Red indicates highly conserved residues, and blue indicates less conserved ones. The default COBALT parameters as implemented in the NCBI server were applied to the following full-length sequences: (1) Chlamydia trachomatis gi815047186 CopN, (2) Chlamydophila pneumoniae TW-183 gi33236178 CopN, (3) Dickeya chrysanthemi gi28628125 HrpJ, (4) Escherichia coli O157:H7 strain EC1212 gi320191267 SepL, (5) Edwardsiella tarda gi62199637 EsaL, (6) Erwinia amylovora gi490258129 HrpJ, (7) Erwinia pyrifoliae gi76152309 HrpJ, (8) Pseudomonas aeruginosa gi553773560 PopN, (9) Pseudomonas syringae pv. phaseolicola 1448A gi71555894 HrpJ, (10) Pantoea stewartii gi727284548 HrpJ, (11) Pectobacterium atrosepticum gi42560417 HrpJ, (12) Pectobacterium carotovorum subsp. carotovorum gi34500870 HrpJ, (13) Pseudomonas cichorii gi182440964 HrpJ, (14) Pseudomonas fluorescens gi985769371 RspJ, (15) Salmonella enterica subsp. enterica serovar Typhimurium gi16766203 InvE, (16) Salmonella enterica subsp. enterica serovar Gallinarum gi309243400 SsaL, (17) Shigella flexneri 2a gi874339429 MxiC, (18) Shigella sonnei Ss046 gi73858422 MxiC, and (19) Yersinia enterocolitica W22703 gi10955559 YopN. The Lilic et al. (18) β-motifs are marked. Only the second β-motif, which binds to the YscB-like chaperones and neighboring area, is conserved and easily identifiable. P. savastanoi is an alternate name for P. syringae. (B) Multiple sequence alignment of TyeA-like domains using conserved domain and local sequence similarity information with COBALT. Aligned sequence stretches with no gaps are colored in blue or red. The red color indicates high sequence conservation; blue indicates lower conservation. Default COBALT parameters were applied to the following full-length sequences: (1) Chlamydia trachomatis gi815047186 CopN, (2) Chlamydophila pneumoniae TW-183 gi33236178 CopN, (3) Dickeya chrysanthemi gi28628125 HrpJ, (4) Escherichia coli O157:H7 strain EC1212 SepL, (5) Edwardsiella tarda gi62199637 EsaL, (6) Erwinia amylovora gi490258129 HrpJ, (7) Erwinia pyrifoliae gi76152309 HrpJ, (8) Pseudomonas aeruginosa gi553773560 PopN, (9) Pseudomonas syringae pv. phaseolicola 1448A gi71555894 HrpJ, (10) Pantoea stewartii gi727284548 HrpJ, (11) Pectobacterium atrosepticum gi42560417 HrpJ, (12) Pectobacterium carotovorum subsp. carotovorum gi34500870 HrpJ, (13) Pseudomonas cichorii gi182440964 HrpJ, (14) Pseudomonas fluorescens gi985769371 RspJ, (15) Salmonella enterica subsp. enterica serovar Typhimurium gi16766203 InvE LT2 (SGSC 1412, ATCC 700720), (16) Salmonella enterica subsp. enterica serovar Gallinarum gi309243400 SsaL, (17) Shigella flexneri 2a gi874339429 MxiC 24570, (18) Shigella sonnei Ss046 gi73858422 MxiC Ss046, and (19) Yersinia enterocolitica AAK69221.1 TyeA. Download FIG S1, TIF file, 2.4 MB. Copyright © 2018 Charova et al. 2018 Charova et al. This content is distributed under the terms of the Creative Commons Attribution 4.0 International license.

**FIG 1:**
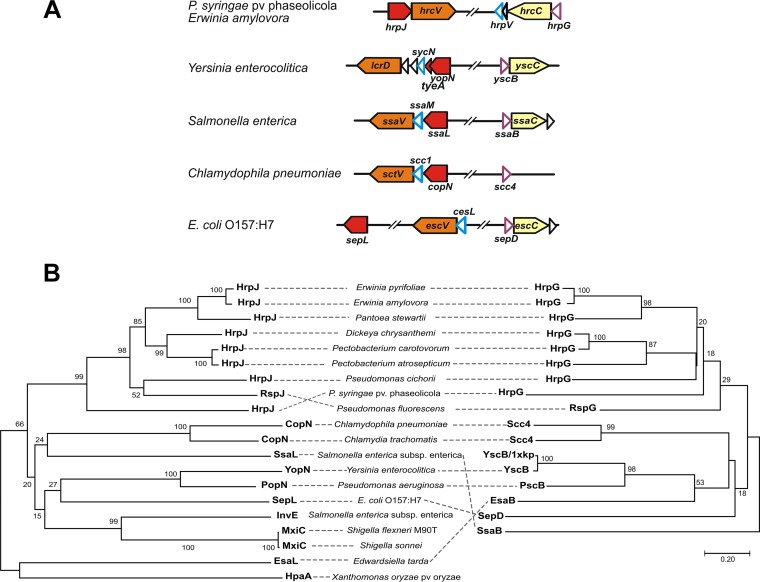
Sequence and synteny analyses establish for HrpG the additional role of a subunit of a class I chaperone for HrpJ. (A) Genetic organization of T3SS gene cluster regions encoding gatekeeper-specific chaperones in various bacteria. The C-terminal region of HrpJ, SsaL, SepL, and CopN proteins is homologous to TyeA of Yersinia enterocolitica (the corresponding genes are depicted in red). The genes *hrpG*, *ssaB*, *sepD*, and *yscB* coding for the first subunit of the gatekeeper chaperone (purple outlines) are located always upstream of the gene coding for the T3SS secretin (light yellow), with the exception of *Chlamydia*, where the secretin gene is lost (54, 55), but the gene organization resembles that of Salmonella enterica. The position of the gene coding for the second subunit of the gatekeeper chaperone (cyan outline) is more variable. (B) Phylogenetic tree of the T3SS gatekeeper proteins juxtaposed with the phylogenetic tree of their cognate chaperones. The phylogenetic relations were inferred using the neighbor-joining method (56), the bootstrap values are shown next to the branches (57), and evolutionary distances were computed using the Poisson correction method (58). Analyses were performed with MEGA7 software (59).

Syntenic analyses of T3SS gene clusters (Fig. 1A and S2) reveal that the *yscB* gene and its counterparts, which encode gatekeeper-specific chaperones, are located immediately upstream of the secretin-encoding gene, i.e., *yscC*, *hrcC*, etc. (19). Interestingly, in the Hrc-Hrp 1 family this location is occupied by the *hrpG* gene, and therefore, it is possible that the transcriptional regulator HrpG has a role as a class I chaperone for HrpJ, in addition to its known function as a negative regulator of HrpV. The similarity of phylogenetic trees and the symmetry of branches (mirror trees) between HrpG and HrpJ homologues (Fig. 1B) suggest a coevolution pattern of the two proteins in Hrc-Hrp 1 systems, which among other possibilities could provide evidence for an interaction between the two proteins.

10.1128/mBio.01096-18.3 FIG S2  Multiple alignment of class I T3SS chaperone sequences encoded by genes located upstream of the gene coding for the T3SS secretin. The PROMALS3D server was used to align known HrpG sequences together with sequences encoded by syntenic loci from animal-pathogenic bacteria. For the alignment, the known YscB structure (chain C of the 1XKP entry from the Protein Data Bank) was used along with GenBank sequences WP_009872035, AAD18852.1, AAC31974.1, EFW65902.1, AAB49178.1, ABA39799.1, AAC44773.1, AAZ33082.1, AAG01462.2, AAS20365.1, AAQ73914.1, BAG24122.1, AMD40287.1, NP_460358.1, and P0C2M8.1. Each residue is colored according to PSIPRED^13^ secondary structure predictions (red, α-helix; blue, β-strand). Consensus secondary structure: pink, α-helix; light blue, β-strand. Download FIG S2, TIF file, 2.1 MB. Copyright © 2018 Charova et al. 2018 Charova et al. This content is distributed under the terms of the Creative Commons Attribution 4.0 International license.

### HrpG, HrpV, and HrpJ form the gatekeeper complex in *P. syringae* pv. phaseolicola and *E. amylovora*.

**FIG 2:**
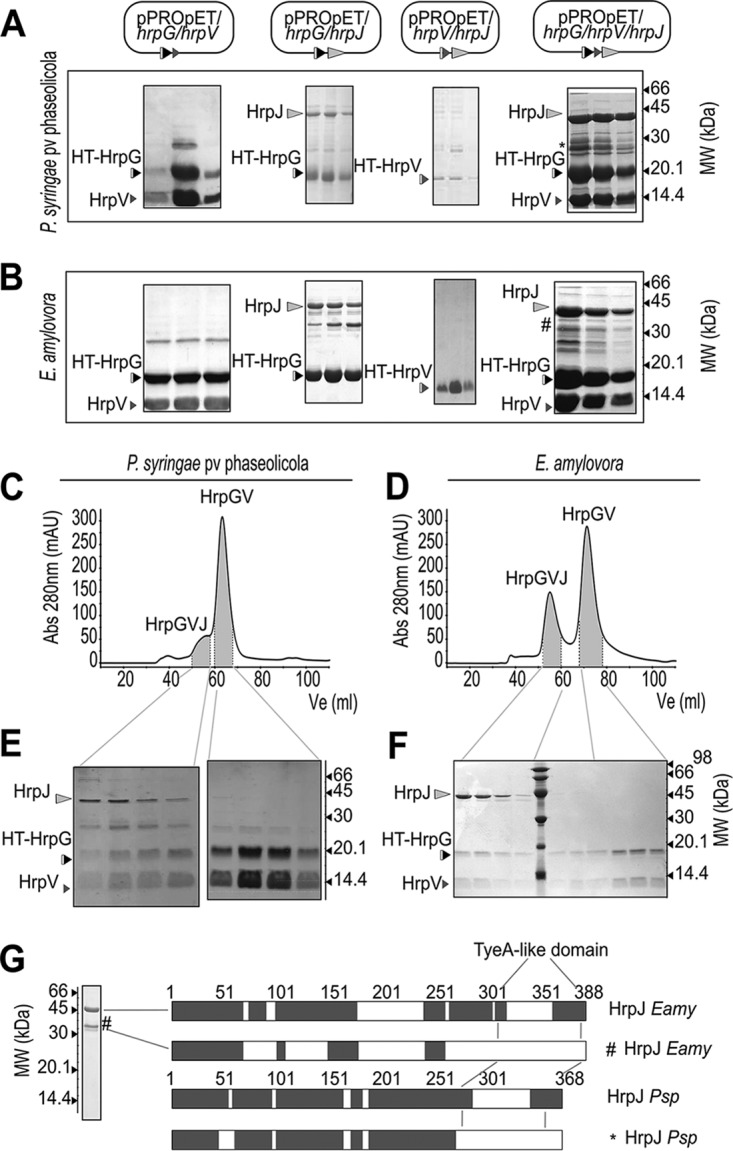
HrpG, HrpV, and HrpJ form the gatekeeper complex in *P. syringae* pv. phaseolicola and *E. amylovora*. (A and B) Successive elutions derived from affinity chromatography isolation of various E. coli-expressed complexes between the HrpG, HrpV, and HrpJ proteins from *P. syringae* pv. phaseolicola and *E. amylovora* analyzed by SDS-PAGE. His_6_-tagged proteins are designated by HT. (C to F) SEC of HrpG/HrpV/HrpJ from *P. syringae* pv. phaseolicola and *E. amylovora* showing the ternary HrpG/HrpV/HrpJ complex coexisting with the HrpG/HrpV complex. The estimated molecular weights of the HrpG/HrpV/HrpJ and HrpG/HrpV complexes from *P. syringae* pv. phaseolicola are 72 kDa and 32 kDa, respectively, while in *E. amylovora* HrpG/HrpV/HrpJ elutes at 74 kDa and HrpG/HrpV elutes abnormally at 18 kDa. (G) nLC-MS/MS identification of full-length and cleaved forms of HrpJ from *E. amylovora* and *P. syringae* pv. phaseolicola. Due to solubility problems, HrpJ from *P. syringae* pv. phaseolicola was analyzed from the soluble HrpG/HrpV/HrpJ complex. The truncated versions of HrpJ are depicted with the symbols * and #, respectively, in panels A, B, and G.

To explore possible interactions hinted at by the *in silico* findings, various binary protein complexes, each comprising a different combination of HrpG, HrpV, and HrpJ, were isolated by affinity chromatography after heterologous overexpression of *P. syringae* pv. phaseolicola or *E. amylovora* genes in Escherichia coli host cells and characterized. The complexes of HrpG/HrpV and HrpG/HrpJ were purified, while the HrpV/HrpJ complex could not be isolated (Fig. 2A and B). As HrpG and HrpV are the only proteins from the Hrc-Hrp 1 pathogenicity island with a predicted class I chaperone fold, this observation fits the *in silico* analysis of T3SS gene clusters (Fig. 1A), further suggesting that the HrpG/HrpV complex may function as a heterodimeric chaperone for the gatekeeper HrpJ, in addition to the known roles of the two proteins in transcription regulation (6, 9).

Possible interactions of HrpJ with the HrpG/HrpV complex were thus investigated via polycistronic constructions of the *P. syringae* pv. phaseolicola or *E. amylovora* genes which were expressed in E. coli host strains. Using affinity chromatography, a soluble ternary complex of HrpG/HrpV/HrpJ was isolated. Interestingly, the three proteins become soluble in the context of the complex, which strongly contrasts with the behavior of, e.g., HrpG from *E. amylovora* and HrpJ from *P. syringae* pv. phaseolicola, which are insoluble when expressed alone. The identity of all interacting proteins was verified by mass spectrometry (MS)-based bottom-up proteomic analysis (Fig. 2G and S3). Interestingly, recombinant HrpJ is accompanied by an additional, truncated form lacking the C-terminal domain (Fig. 2G). This truncated HrpJ form is less abundant in the context of the triple complex (Fig. 2A and B), possibly reflecting an additional function for the complex, i.e., that of HrpJ stabilization, in both *P. syringae* pv. phaseolicola and *E. amylovora*.

10.1128/mBio.01096-18.4 FIG S3  MS-based identification of HrpG, HrpV, and HrpJ. nLC-MS/MS identification of HrpG, HrpV, and HrpJ from *P. syringae* pv. phaseolicola and *E. amylovora* derived from affinity chromatography purification of the triple HrpG/HrpV/HrpJ complexes (Fig. 2A and B). In gray, yellow, and red are shown the identified regions of the proteins with high, medium, and low probability, respectively. Download FIG S3, TIF file, 1.4 MB. Copyright © 2018 Charova et al. 2018 Charova et al. This content is distributed under the terms of the Creative Commons Attribution 4.0 International license.

### HrpG, HrpV, and HrpJ form a 1:1:1 triple complex in solution.

Size exclusion chromatography (SEC) analysis of the heterologously expressed HrpG/HrpV/HrpJ complex from *P. syringae* pv. phaseolicola and *E. amylovora* revealed in both cases the coexistence of two distinct populations, one of which corresponds to the triple complex HrpG/HrpV/HrpJ and the other of which corresponds to the HrpG/HrpV heterodimer (Fig. 2C to F).

**FIG 3:**
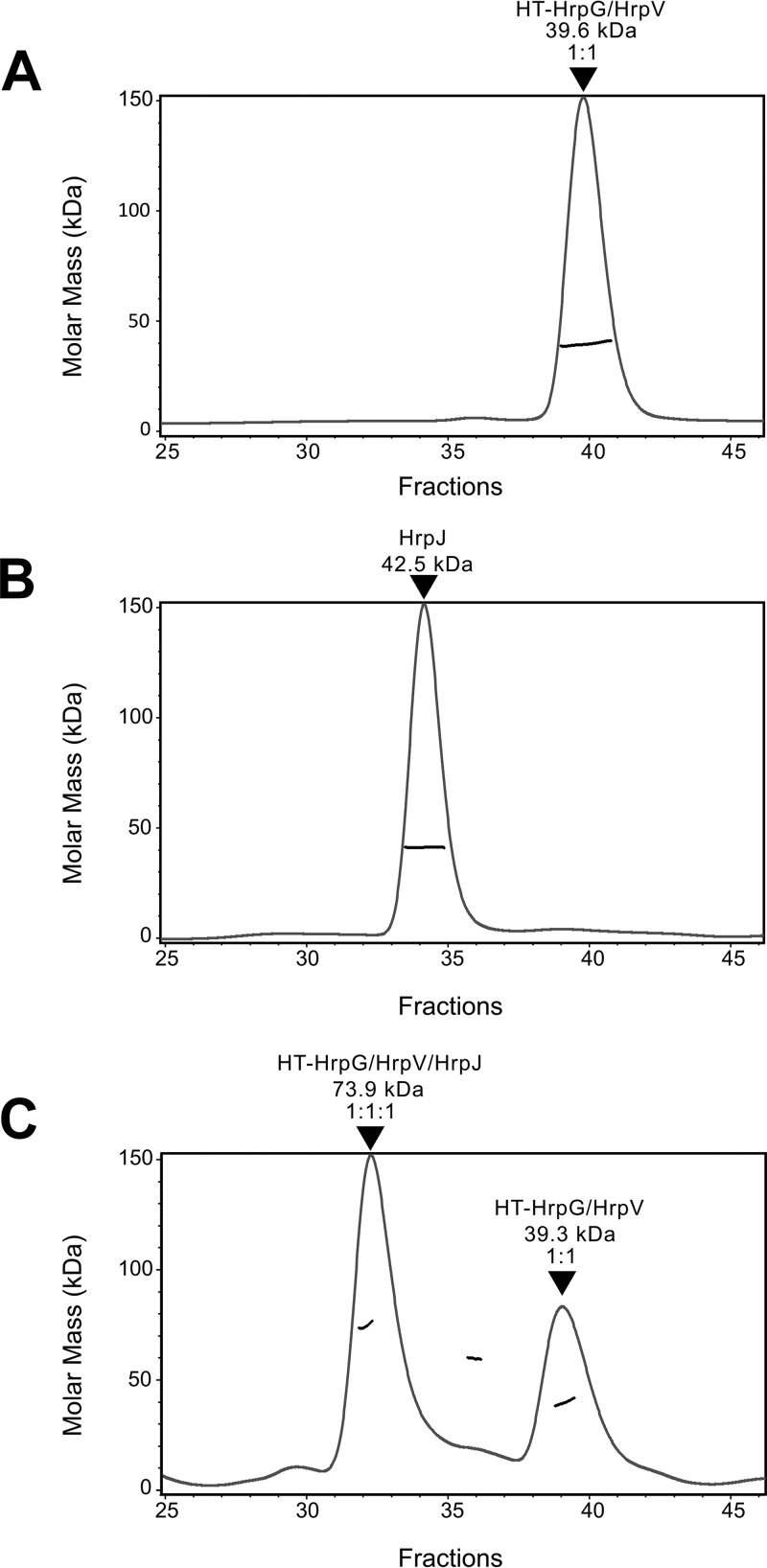
HrpG, HrpV, and HrpJ form a 1:1:1 triple complex in solution. Separately purified HrpG/HrpV complex (A) and HrpJ (B) were run on a Superdex 200 column, giving rise to peaks corresponding to calculated masses of 39.6 kDa and 42.5 kDa, respectively. Following overnight coincubation (41 nmol of the HrpG/HrpV complex and 35 nmol of HrpJ), a sample from the mixture was run on the same column, giving rise to two distinct peaks (C), corresponding to the reconstituted HrpG/HrpV/HrpJ complex with a calculated mass of 73.9 kDa and to the HrpG/HrpV complex with a mass of 39.3 kDa. The HrpG/HrpV peak shows a slight, buffer-dependent shift.

To further characterize the formation of the triple complex, the HrpG/HrpV complex and HrpJ from *E. amylovora* were separately produced and incubated overnight at an approximately 1:1 molar ratio. The association of HrpJ and HrpG/HrpV was monitored using an SEC column coupled to a multiangle laser light scattering (MALLS) detector (Fig. 3A and B). The HrpG/HrpV/HrpJ complex was detected with a calculated molecular mass of 74 kDa, corresponding to a stoichiometry of 1:1:1 among the three proteins. An additional, smaller population of the HrpG/HrpV complex was also detected (Fig. 3C).

### The three-dimensional (3D) structure of the HrpG/HrpV/HrpJ complex of *E. amylovora* resembles a T3SS gatekeeper complex.

**FIG 4:**
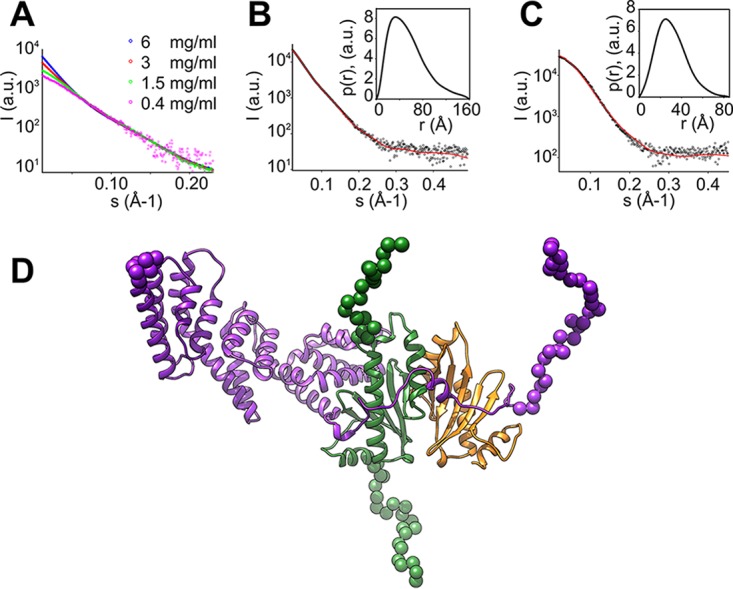
The 3D structure of the HrpG/HrpV/HrpJ complex of *E. amylovora* resembles a T3SS gatekeeper complex. (A) Effect of concentration on the low angle region of the SAXS data of the HrpG/HrpV/HrpJ complex. (B and C) Experimental SAXS patterns of the HrpG/HrpV/HrpJ complex (B) and of the HrpG/HrpV subcomplex (C), shown with black circles, with the corresponding fits of the model to the experimental data shown in red lines; the insets show the pair distance distribution functions *p*(*r*). a.u., absolute units. (D) Model of the HrpG/HrpV/HrpJ complex with HrpG in green, HrpV in orange, and HrpJ in purple; dummy residues are shown as spheres.

Small-angle X-ray scattering (SAXS) studies of the *E. amylovora* HrpG/HrpV/HrpJ complex reveal a high propensity for (reversible) aggregation as evidenced by the increasing intensity at lower angles (Fig. 4A) with increasing concentration. Moreover, the complex dissociates at very low concentrations. This is evident from the molecular mass values estimated from both the Guinier (20) approximation (55 kDa) and the Porod (21) volume (60 kDa). For this purpose, the scattering data obtained at an intermediate concentration (1.5 mg/ml) with the molecular mass calculated from the Guinier plot (75 kDa) and from the Porod volume (78 kDa) and a radius of gyration (*R*_*g*_) of 40 Å were used as a compromise between excessive aggregation and dissociation. The pair distance distribution function *p*(*r*) (22) shows an elongated structure with a maximum particle dimension (Dmax) of 160 Å (Fig. 4B, inset), in contrast to the more spherical structure of the HrpG/HrpV subcomplex that we have characterized in our previous work (11) (Fig. 4C, inset).

Despite low sequence identity (Fig. S1 and S2), we used the only available structure of a gatekeeper complex in the analysis of SAXS data. In the Y. pestis complex, YscB/SycN/TyeA/YopN, the counterpart of HrpJ, consists of two polypeptide chains, TyeA and YopN; the structure of the complete YscB/SycN/TyeA/YopN complex was derived from the combination of two separate crystal structures, with the Protein Data Bank codes 1XKP and 1XL3 (17). HHpred fold recognition (23) indicates that the complexes HrpG/HrpV/HrpJ and YscB/SycN/TyeA/YopN potentially share the same overall fold, with the transcriptional regulators HrpG and HrpV from the *E. amylovora* T3SS corresponding to the gatekeeper chaperones YscB and SycN from Y. pestis, respectively. We constructed homology models for all proteins and arranged them similarly to the YscB/SycN/TyeA/YopN complex structure with similar pairwise interactions. To compare them with the experimental SAXS data, the program CORAL (24) was used to add dummy residues that represent the excess electron density of the residues not present in the homology models. One such model is shown in Fig. 4D. The fit to the experimental data is satisfactory (Fig. 4B), indicating that the HrpG/HrpV/HrpJ complex from the phytopathogenic T3SS has a structure very similar to the YscB/SycN/TyeA/YopN complex from the T3SS of Y. pestis. The fit of SAXS data (11) from the HrpG/HrpV complex to the respective part of the model is not as good (Fig. 4C), which may be attributed to conformational changes when the trimeric complex is formed. Overall, however, this analysis strongly suggests that the solution structure of the *E. amylovora* HrpG/HrpV/HrpJ complex is indeed a gatekeeper complex.

### The HrpG-HrcU interaction suggests that the HrpG/HrpV/HrpJ complex is docked to the bacterial membrane.

**FIG 5:**
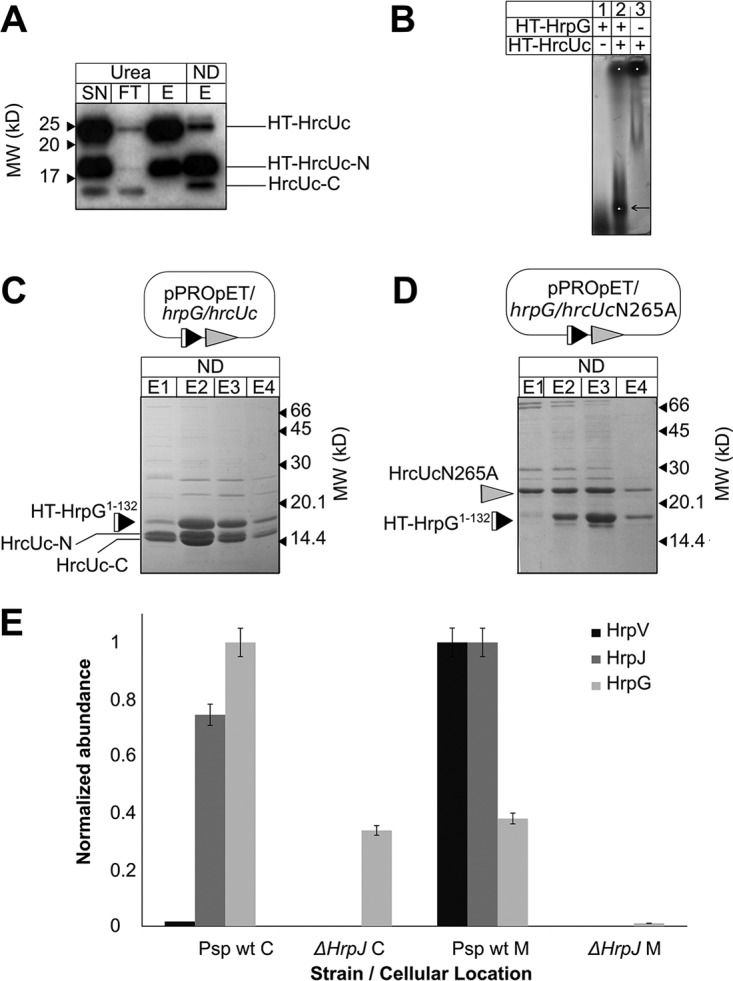
The *P. syringae* pv. phaseolicola HrpG/HrpV/HrpJ complex is docked to the bacterial membrane. (A) Western detection of C-terminal *P. syringae* pv. phaseolicola HrcU (HrcUc, residues 199 to 359) fragments from affinity chromatography samples under nondenaturing and denaturing (8 M urea) conditions using a specific polyclonal antibody for HrcU. HT-HrcUc is self-cleaved, and the untagged C-terminal cleavage fragment (HrcUc-C) corresponding to residues 267 to 359 copurifies with the tagged fragments under nondenaturing conditions. Urea treatment separates the HrcUc fragments. Self-cleavage is partial, leaving an amount of uncleaved protein. (B) A fraction of *P. syringae* pv. phaseolicola HrpG comigrates with HrcUc in native agarose gel electrophoresis (arrow). (C) *P. syringae* pv. phaseolicola HT-HrpG^1–132^ copurifies with HrcUc fragments after coexpression. (D) Coexpression and copurification of *P. syringae* pv. phaseolicola HT-HrpG^1–132^ with the cleavage-deficient HrcUc mutant N265A. The uncleaved HrcUc still interacts with HT-HrpG^1–132^. (E) Distribution of *P. syringae* pv. phaseolicola HrpG, HrpV, and HrpJ proteins in the cytosol and membrane fractions of wild-type and Δ*hrpJ P. syringae* pv. phaseolicola as determined through nLC-MS/MS analysis. The normalized abundances of the three proteins from wild type (wt) and Δ*hrpJ* mutant in *P. syringae* pv. phaseolicola cytosol (C) and membranes (M) show a significant HrpJ-dependent enrichment of the HrpG/HrpV/HrpJ complex in membranes. Lane labels: SN, supernatant after sonication and centrifugation; FT, column flowthrough; E (E1, E2, E3, and E4), elution fractions; ND, nondenaturing conditions; Urea, denaturing conditions.

The cytoplasmic conserved domain of inner membrane-associated T3SS proteins from the SctU family (FlhB, YscU, EscU, etc.) has been observed to undergo a highly specific self-cleavage, which plays a role in T3SS regulation. After cleavage, the two fragments continue to interact (25). We observed a similar behavior for the *P. syringae* pv. phaseolicola family member HrcU (Fig. 5A); self-cleavage of the heterologously produced 21-kDa cytoplasmic C-terminal domain of HrcU produces two interacting domains of 11 and 10 kDa. The process is partial, leaving a fraction of the protein uncleaved.

For the C-terminal domain of *P. syringae* pv. phaseolicola HrcU, we observed a binding interaction with HrpG, since the two proteins comigrate in native agarose gels (Fig. 5B) and copurify when coexpressed (Fig. 5C and S4). However, self-cleavage is not important for this interaction, as a cleavage-resistant HrcU variant carrying a mutation (N265A) in the specific self-cleavage (26) sequence (NPTH) of the protein still interacts with HrpG (Fig. 5D). A complex of HrpG with C-terminal HrcU could not be isolated in *E. amylovora*, although the presence of HrcU greatly improved the solubility of the otherwise insoluble HrpG protein.

10.1128/mBio.01096-18.5 FIG S4  Comparative denaturing and nondenaturing purifications of *P. syringae* pv. phaseolicola HrpG^1–132^ coexpressed with proteolytic fragments of HrcUc. Coomassie blue-stained SDS-PAGE gels with affinity chromatography fractions containing His-tagged HrpG and HrcUc under nondenaturing (ND) and denaturing (urea) conditions. (A) The elution fraction (E) includes bands HrcUc-N and HrcUc-C, corresponding to the proteolytic fragments after self-cleavage. The band corresponding to the uncleaved C-terminal domain (HrcUc) is also present. (B) Under denaturing conditions, only the band corresponding to His-tagged HrpG^1–132^ is observed in the elution fraction. SN, supernatant; P, pellet; FT, flowthrough fraction. Download FIG S4, TIF file, 0.7 MB. Copyright © 2018 Charova et al. 2018 Charova et al. This content is distributed under the terms of the Creative Commons Attribution 4.0 International license.

The binding of HrpG to HrcU implies, therefore, that the HrpG/HrpV/HrpJ complex is probably located in the proximity of the inner bacterial membrane. This hypothesis is supported by localization experiments performed in *P. syringae* pv. phaseolicola using MS analysis, which confirm that HrpG, HrpV, and HrpJ colocalize predominantly at the bacterial membrane (Fig. 5E). Furthermore, the anchoring of HrpG/HrpV/HrpJ to the membrane is HrpJ dependent, as suggested by the lack of HrpG or HrpV enrichment in the membrane fractions of an Δ*hrpJ* knockout mutant. This behavior could reflect a critical contribution of HrpJ to the stabilization of the interaction between the gatekeeper complex and HrcU.

### In *P. syringae* pv. phaseolicola, the HrpG/HrpV/HrpJ complex orchestrates intermediate T3SS substrate secretion, without affecting early substrates.

To assess the functional implications of the HrpG/HrpV/HrpJ complex, we investigated the secretion of three substrates, i.e., the gatekeeper HrpJ itself; the HrpA2 pilin protein, an early substrate (12); and the harpin HrpZ1, an intermediate substrate.

**FIG 6:**
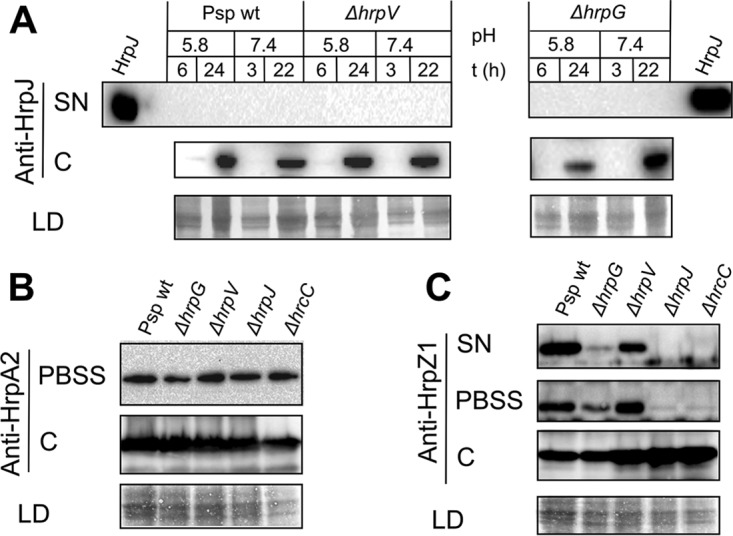
In *P. syringae* pv. phaseolicola T3SS, HrpG/HrpV/HrpJ orchestrates intermediate substrate secretion, without affecting early substrates. Western blots with HrpJ-, HrpA2-, and HrpZ1-specific polyclonal antibodies. (A) HrpJ is not secreted in the medium after *P. syringae* pv. phaseolicola T3SS induction in culture at pH 5.8 (SN) and accumulates late in the course of time in the cell fraction C. Shifting the pH from 5.8 to 7.4 after 6 h of induction does not elicit HrpJ secretion. (B) Most of the HrpA2 protein precipitates with the cell fraction after centrifugation in PBSS with the total levels not differing substantially between wild-type and mutant *P. syringae* pv. phaseolicola strains. The Δ*hrcC* mutant (negative secretion control) still secretes HrpA2 at wild-type levels, as reported previously (30). (C) Secretion and accumulation of *P. syringae* pv. phaseolicola HrpZ1 are severely reduced in the Δ*hrpG* mutant, the Δ*hrpV* mutant secretes reduced amounts of HrpZ1 compared to the wild-type *P. syringae* pv. phaseolicola, and the Δ*hrpJ* mutant accumulates but does not secrete any HrpZ1, resembling in both aspects the secretion-incompetent Δ*hrcC* mutant. The PBSS treatment does not result in cell lysis as deduced from HrpZ1 levels of the Δ*hrcC* mutant. Abbreviations: SN, concentrated culture supernatant; PBSS, PBS plus 0.1% SDS outer extract from intact cells; C, cell fraction; LD, SDS-PAGE of the total protein loaded in cell fraction C shown for normalization purposes.

In contrast to P. syringae pv. tomato DC3000 and *E. amylovora*, where HrpJ is found in culture supernatants after induction of T3SS under laboratory conditions (15, 27), we did not detect endogenous HrpJ in the secreted fraction, either in wild-type *P. syringae* pv. phaseolicola or in the Δ*hrpG* and Δ*hrpV* knockout mutants (Fig. 6A). HrpJ secretion is furthermore not affected by pH, unlike the *Salmonella* T3SS, where a pH shift is a signal for secretion (28). Additionally, it accumulates late in the course of time, in line with the recent literature in which induction of the *hrpJ* operon is suppressed until high-enough levels of HrpL are expressed (29).

On the other hand, the detection of HrpA2 pilin showed quantitative inconsistencies, as also observed in previous studies (15, 16). These may be attributed to pilin aggregation and coprecipitation with the cellular fractions. This problem was overcome by applying a mild sodium dodecyl sulfate (SDS) treatment (0.01% in phosphate-buffered saline [PBS], pH 7.4, here called PBSS extract) of the precipitated cells, after which most of the extracellular HrpA2 pilin was found in the PBSS extract (Fig. 6B). Approximately equal amounts of HrpA2 were detected for all *P. syringae* pv. phaseolicola strains tested (either wild type or Δ*hrpG*, Δ*hrpV*, and Δ*hrpJ* knockout mutants). Interestingly, HrpA2 pilin was also found in the PBSS extract originating from a secretion-incompetent Δ*hrcC* mutant. Since no HrpZ1 was found in this case, we can rule out cell disruption as a cause for these observations (Fig. 6B). Since observations of T3SS-independent HrpA2 secretion (see Fig. 6 in reference 30) exist in the literature, though not discussed in any detail, our experiments suggest that in the absence of a functional T3SS secretory pore (Δ*hrcC* mutant strains are considered incapable of type III secretion), the accumulated HrpA2 may be exported via an unidentified, alternative, T3SS-independent pathway, possibly in order to maintain cell viability and homeostasis. It is noteworthy that flagellin can be exported from the T3SS (31), so one could hypothesize that the reverse may also be true, i.e., HrpA2 may be exported by the flagellum. Additionally, we have reported earlier the presence of a second, constitutively expressed rhizobium-like T3SS in *P. syringae* pv. phaseolicola (32) that may act as a conduit for HrpA2 secretion when the main secretion pore becomes unavailable. In summary, in *P. syringae* pv. phaseolicola the HrpG, HrpV, and HrpJ proteins have no influence in HrpA2 accumulation and secretion.

In contrast to the unaltered secretion profile of HrpA2, HrpZ1 showed dramatic differences in its extracellular and intracellular detection for wild-type *P. syringae* pv. phaseolicola and the Δ*hrpG*, Δ*hrpV*, or Δ*hrpJ* mutants (Fig. 6C). Most of the HrpZ1 protein is located extracellularly in wild-type *P. syringae* pv. phaseolicola, while the Δ*hrpJ* mutant fails to secrete HrpZ1, similarly to the Δ*hrcC* strain, a secretion-incompetent mutant. The absence of HrpZ1 secretion by a Δ*hrpJ* mutant had already been reported in the literature for *P. syringae* pv. tomato DC3000 (15, 16). The Δ*hrpG* mutant, on the other hand, exhibits a significant reduction in the total amount of HrpZ1 (intracellular and extracellular), reflecting reduced expression levels, as expected for a T3SS which is repressed through the action of HrpV in the absence of HrpG. The severely reduced levels of secreted HrpZ1 could be also caused by reduced gatekeeper activity. A previous report (9) also showed that a Δ*hrpG* mutant of Pseudomonas syringae pv. syringae 61 also fails to accumulate and secrete HrpZ1. On the other hand, the Δ*hrpV* mutant shows a small but reproducible reduction of HrpZ1 secretion (Fig. 6C). Finally, it is noteworthy that the Δ*hrpV* mutant displays increased expression of *hrpL* compared to wild-type *P. syringae* pv. phaseolicola (as expected) in contrast to the *hrpL* reduction observed on the Δ*hrpJ* mutant (Fig. S5). From these results, we conclude that the HrpG/HrpV/HrpJ complex is involved in two events, i.e., in the derepression of the HrpV/HrpS/HrpR circuit and in the promotion of HrpZ1 secretion, probably as a result of the chaperone effects of HrpG/HrpV on HrpJ and formation of the gatekeeper complex.

10.1128/mBio.01096-18.6 FIG S5  Opposing effects of HrpV and HrpJ in transcription of the T3SS-specific alternative *σ* factor HrpL. Expression analysis using quantitative reverse transcription PCR (RT-qPCR) carried out on wild-type (triangles) and Δ*hrpV* (diamonds) and Δ*hrpJ* (circles) mutant *P. syringae* pv. phaseolicola strains grown in Hrp-inducing medium (HIM). Graph shows number of transcripts relative to 16S for *hrpL*. Bars reflect the mean values obtained from triplicate analyses for each strain, error bars represent the standard errors, and symbols indicate the individual values. The statistical significance of the differences, as established by homoscedastic and 2-tailed Student’s *t* test, is marked with asterisks (*, *P* < 0.05; ****, *P* < 0.001). Download FIG S5, TIF file, 0.1 MB. Copyright © 2018 Charova et al. 2018 Charova et al. This content is distributed under the terms of the Creative Commons Attribution 4.0 International license.

## DISCUSSION

**FIG 7:**
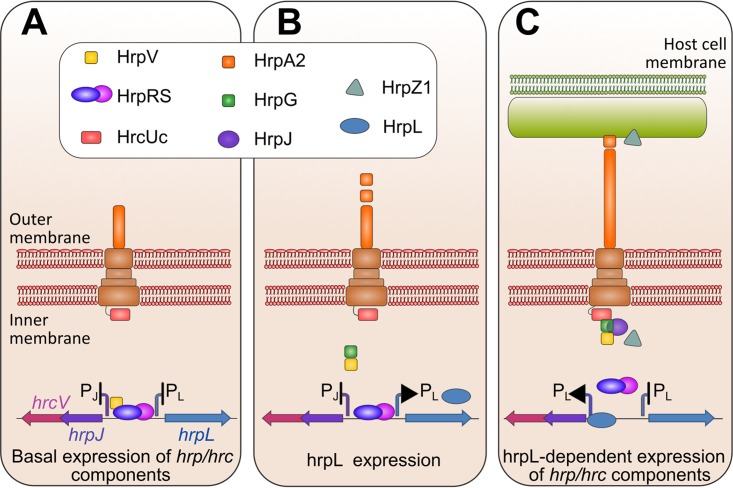
The interaction network coupling T3SS expression regulation to secretion in plant-pathogenic T3SS. (A) Repressed state of T3SS gene expression established through the HrpV action on HrpR/HrpS (6, 60). (B) Formation of the HrpG/HrpV complex and initiation of the derepression of gene expression. (C) Formation of the HrpG/HrpV/HrpJ gatekeeper complex and HrpJ-dependent anchoring of the ternary complex at the bacterial membranes, possibly via a HrpG/HrcUc binding. HrpS derepression is completed, and intermediate substrate secretion is allowed.

Our findings provide the basis for a model of T3SS activation, through the discovery of protein-protein interaction networks linking two key T3SS processes, gene expression control and intermediate substrate secretion (Fig. 7). A central role in this interaction network is played by three proteins, HrpG, HrpV, and HrpJ. Experimental evidence from *P. syringae* pv. phaseolicola and *E. amylovora* suggests that in phytopathogenic bacteria, the HrpG and HrpV proteins, in addition to their roles in T3SS transcription control, also act as a heterodimeric class I chaperone for the gatekeeper HrpJ, a protein for which no interactions with chaperones were known until now. Synteny analyses and phylogenetic studies along with protein expression, biochemical experiments, and structural studies provide additional support for a chaperone role for the HrpG/HrpV heterodimer in Hrc-Hrp 1 systems, associating with the HrpJ protein and leading to the formation of a ternary gatekeeper complex.

The C-terminal domain of HrpJ is homologous to TyeA, a small T3SS protein from bacteria such as Y. pestis, Vibrio parahaemolyticus, Pseudomonas aeruginosa, Aeromonas salmonicida, Photorhabdus luminescens, etc. (14). The *Yersinia* TyeA protein anchors the gatekeeper YopN to the cytoplasmic side of the T3SS export apparatus, blocking the premature secretion of effectors; upon receipt of a host signal, YopN is secreted, permitting subsequent effector secretion. A naturally occurring YopN/TyeA fusion has been shown to block Yop effector secretion (33). Τhe opposite case which has been observed by us in HrpJ, i.e., cleavage of the naturally occurring, single-chain gatekeeper protein into two modules corresponding to YopN and TyeA (Fig. 2G), has to our knowledge not been reported earlier. Our experiments reveal two forms of HrpJ, i.e., a truncated form lacking the TyeA-like domain and the full-length form of the protein, in both *P. syringae* pv. phaseolicola and *E. amylovora*. In copurification assays, the presence of HrpG/HrpV in the ternary complex appears to reduce the cleavage of the C-terminal domain, stabilizing full-length HrpJ (Fig. 2A, B, and G). Whether this cleavage occurs also *in vivo* and whether it is physiologically significant remain to be determined.

Gatekeepers are docked to the cytoplasmic side of the T3SS export channel, where they exert their role as plugs blocking premature effector secretion (28, 34–36). In this work, we have identified binding of the *P. syringae* pv. phaseolicola HrpG with the cytoplasmic domain of HrcU, an inner membrane core component. The HrpG-HrcU interaction, which occurs both in the self-cleaved and in the uncleaved form of HrcU, along with our data from native localization experiments (Fig. 5), reveal that the HrpG/HrpV/HrpJ complex resides near *P. syringae* pv. phaseolicola membranes. The anchoring of the complex to the membranes is HrpJ dependent and occurs possibly via a HrpG-HrcU interaction. Thus, the docking of the gatekeeper complex to the membranes is a critical step, via which the HrpG and HrpV proteins are potentially removed from the proximity of DNA and migrate toward the inner bacterial membrane. HrpG is capable of relieving only part of the HrpV-mediated repression (as would be expected for proteins expressed by the same operon) of T3SS transcription factors HrpR/HrpS (6). Our findings open the possibility that recruitment of HrpG/HrpV by HrpJ and anchoring to the membrane of the bacterium contribute to the derepression of the T3SS expression. This hypothesis is supported by the reduced expression of *hrpL* in the Δ*hrpJ* mutant (see Fig. S5 in the supplemental material). From its new position, the assembled ternary complex can exert its role on substrate secretion. The observed interactions of HrpG with the gatekeeper and the bacterial membranes are in line with the highly interactive nature of this protein, for which the binding to HrpF (37), an additional negative regulator of the system and a pilin-stabilizing component, has been reported recently. Earlier studies in *P. syringae* pv. tomato have shown that HrpJ is secreted in culture after T3SS induction; its secretion is not a prerequisite, however, for harpin secretion regulation (16), while HrpJ from *E. amylovora* is also secreted under inducing conditions (27). In this work, we have shown that in *P. syringae* pv. phaseolicola the HrpJ protein is not secreted under culture conditions and positively controls the secretion of harpin HrpZ1, an intermediate T3SS substrate, while not affecting HrpA2, an early secretion substrate (Fig. 6). Moreover, knockout mutants of HrpG and HrpV do not affect secretion of HrpA2, but they display changes in the levels of accumulated and secreted HrpZ1. Beyond its effects on harpins, a possible role of the HrpG/HrpV/HrpJ complex on controlling secretion and/or accumulation of T3SS effectors needs to be further investigated.

HrpV and HrpG are key T3SS components forming an antiactivator-antiantiactivator pair that regulates T3SS transcription (9). Although HrpV from *E. amylovora* has a low sequence identity (17%) to the *P. syringae* pv. phaseolicola HrpV, the existence of the HrpG/HrpV and HrpG/HrpV/HrpJ complexes in both bacterial species strongly suggests a common regulation of Hrc-Hrp 1 systems, in which the proteins HrpG, HrpV, HrpS, and HrpJ form a dynamic circuit responsible for fine-tuning transcription and secretion. This functional coupling is a novel concept for phytopathogenic systems. Examples from interaction analyses that remotely point to a comparable functional coupling in T3SS can be found in animal-pathogenic bacteria (38–40) with the SepL, SepD, and CesL proteins from enterohemorrhagic and enteropathogenic E. coli (41) representing the closest case to our observations. In this system, SepL, a gatekeeper protein, interacts with SepD and CesL, with the latter also having an effect on transcription. The present analysis is the first one to accentuate a possible mechanistic coupling that is realized via migration of T3SS components between subcellular compartments. It is not unreasonable to expect that similar couplings, not yet identified, exist in other pathogens.

Furthermore, in *P. syringae* pv. phaseolicola the HrpV/HrpG double-negative regulatory loop is responsible for the stochastic establishment of phenotypically distinct subpopulations differing in the expression of the T3SS (10). The results presented here add two novel aspects to our current knowledge on phenotypic heterogeneity within clonal populations: (i) they provide a direct link between the bistability of gene expression and the bistability of the secretion phenotype, and (ii) by proposing distinct cellular locations for HrpV/HrpG, one proximal to DNA and additionally a membrane-associated one, our results have implications on how these two proteins may be distributed between dividing cells, an important aspect determining switching between ON and OFF states at the single-cell level during cell division.

The circuit presented here fills an important gap in our understanding of the complicated yet elegant network of the regulatory interactions occurring during phytopathogenic T3SS activation. The discovery of transcription-secretion coupling in remotely related pathogens suggests that the confluence of T3SS pathways through component migration might reflect a general and important mechanism in T3SS activation. The junction point between these pathways probably represents an attractive target (in addition to the exposed extracellular pilus components) for the development of antibacterial strategies affecting both the expression and secretion cascades of T3SS.

## MATERIALS AND METHODS

### Protein production and purification.

E. coli BL21(DE3) cells transformed with the pPROpET recombinant constructions bearing combinations of *hrpG*, *hrpV*, *hrpJ*, and *hrpG*^*1–132*^/*hrcU*^*199–359*^ (*P. syringae* pv. phaseolicola) or *hrcU*^*199–360*^ (*E. amylovora*) genes from the two plant pathogens of this study were induced using a standard isopropyl-β-d-1-thiogalactopyranoside (IPTG)-based protocol. Overnight saturated Luria-Bertani (LB) cultures were diluted 1:20 in fresh LB, with 50 μg/ml kanamycin and 0.2% glucose, and were grown at 37°C until an optical density at 600 nm (OD_600_) of 0.6 to 0.8 was reached. IPTG was subsequently added to a final concentration of 0.3 mM to each culture, and recombinant protein induction was performed at 23°C for 4 h. The induced cells were precipitated and, for *P. syringae* pv. phaseolicola proteins, were resuspended in 100 ml lysis buffer per liter of induced culture, containing 20 mM Tris (pH 8.0), 50 mM NaCl, 10 mM imidazole, 10 mM 2-mercaptoethanol, 10% glycerol, 0.1% Triton X-100, supplied with 1 mM phenylmethanesulfonyl fluoride (PMSF); for *E. amylovora* proteins, cells were resuspended in 100 ml lysis buffer per liter of induced culture consisting of 20 mM Tris, pH 8.0, 150 mM NaCl, 5 mM imidazole, 5% glycerol, 0.05 mM EDTA, and 10 mM β-mercaptoethanol. Cells were disrupted with sonication in ice, 14 sonication cycles of 30 s each, with cooling intervals of 30 s. The suspension was centrifuged at 18,000 × *g* for 45 min at 4°C. The supernatants were loaded onto small plastic chromatography columns (Bio-Rad) containing 1 ml nickel-nitrilotriacetic acid (Ni-NTA) agarose (Qiagen), preequilibrated with 10 volumes of the corresponding lysis buffer. Three washes were subsequently applied to the column with buffers containing a gradually increasing imidazole concentration. The complexes were eluted from the column at a concentration of 300 mM imidazole.

### T3SS sample preparation for Western blotting.

After T3SS induction in culture, twenty-five milliliters per culture was processed. Cells were precipitated, the supernatant was filtered with 0.22-µm filters, PMSF was added to a final concentration of 1 mM, and the supernatant was processed further using a pyrogallol red-molybdate-methanol (PRMM)-employing protocol (42). The precipitated cells were subjected to a mild treatment with 0.4 ml of PBSS, for 10 min at room temperature, followed by centrifugation. The supernatants were transferred to microcentrifuge tubes and filtered with 0.22-µm filters, 3 volumes of ice-cold acetone was added, and samples were incubated overnight at −20°C. The samples were subsequently centrifuged at 4°C, and pellets were washed once with ice-cold acetone, dried from residual acetone, resuspended in appropriate volumes of 2× standard sample buffer (Laemmli), and boiled for 10 min at 95°C. Cells were subsequently resuspended in PBS, pH 7.4, containing 8 M urea and incubated at room temperature for 30 min. An equal volume of 2× sample buffer was added to the samples, and a boiling step at 95°C for 10 min followed. After boiling, the samples were centrifuged at room temperature to precipitate the solid cellular debris.

Twenty-five milliliters of filtered supernatants were treated as described in established protocols (42); in brief, each supernatant was mixed with an equal volume of PRMM buffer, the pH was set to ~2.8, and mixtures were incubated with agitation for 2 h at room temperature, followed by an extra overnight incubation step at 4°C. The samples were subsequently centrifuged for 1 h at 4°C and 12,000 × *g*, the liquid was carefully discarded, and the pellet was washed twice with ice-cold acetone. Finally, the pellet was resuspended in 2× Laemmli buffer and boiled for 10 min at 95°C. Sample normalization before gel loading was performed as follows: from an initial culture with an OD_600_ of 0.3, the loading cell fraction volume used on standard 14% SDS-PAGE gels was 30 µl (out of a total 300 µl of sample volume), and the corresponding volumes of the SDS extracts and the supernatant samples were 20 µl (out of a total 200 µl of sample volume). Standard 14% polyacrylamide gels were run according to SDS-PAGE protocols for Tris-glycine electrophoresis (43), under a constant voltage of 150 V. Prestained molecular weight markers (VI from Roche, Kaleidoscope from Bio-Rad) were included in the runs.

### T3SS sample preparation for proteomic analysis.

Twenty-hour-induced wild-type and *ΔhrpJ P. syringae* pv. phaseolicola cultures, 200 ml each, were centrifuged for 15 min and 8,000 × *g* at 4°C, and cells were resuspended in 40 ml low-salt lysis buffer (50 mM Tris-Cl, pH 8.0, 2 mM MgCl_2_, 5 mM PMSF). The cell suspensions were sonicated in 10 cycles of 30 s each, with cooling intervals of 30 s. The mixtures were then centrifuged for 15 min at 8,000 × *g* at 4°C to precipitate unbroken cells. The supernatant was transferred to ultracentrifugation tubes and fractionated at 210,000 × *g* for 1 h. The membrane fractions were treated as follows: pellets from the first ultracentrifugation step were resuspended in extraction buffer (50 mM Tris-Cl, pH 8.0, 2 mM MgCl_2_, 5 mM PMSF, 1% Triton X-100), incubated for 30 min at 10°C, and ultracentrifuged as described above. The extracted membranes were then diluted to a final 0.1% concentration of Triton X-100 and concentrated with Amicon centrifugal filters with a molecular weight cutoff of 10,000. Protein content in all samples was measured using the Bradford protocol (44). Amounts of 3.5 µg and 4.5 µg from each cytosolic and membrane fraction, respectively, were analyzed on an 8% native polyacrylamide gel at a constant current of 5 mA for 4 h at 4°C. Analyzed protein complexes were subsequently fixed with 30% methanol and 10% acetic acid, washed thoroughly with distilled water, and finally stained with a blue-silver staining solution compatible with nanoscale liquid chromatographic tandem mass spectrometry (nLC-MS/MS) handling.

### SEC and MALLS.

SEC was performed at 20°C using an ÄKTA purifier system (Amersham) and a prepacked Hi-Prep 16/60 Sephacryl S-200 high-resolution column (GE Healthcare). Flow rate was 0.5 ml/min, and elution was monitored at 280 nm. Protein-containing fractions from Ni-NTA isolation were pooled, concentrated to 2.5 mg/ml for HrpG/HrpV/HrpJ from *P. syringae* pv. phaseolicola and 8 mg/ml for HT-HrpG/HrpV/HrpJ from *E. amylovora*, and loaded using a 2-ml loop. The buffer used for analysis of HrpG/HrpV/HrpJ from *P. syringae* pv. phaseolicola consisted of 50 mM Tris-Cl, pH 8.0, 50 mM NaCl, and 0.5 mM EDTA; that for HrpG/HrpV/HrpJ from *E. amylovora* consisted of 50 mM Tris-Cl, pH 8.0, 100 mM NaCl, 2 mM dithiothreitol (DTT), and 0.5 mM EDTA. Fractions of 2 ml were collected and analyzed using SDS-14% PAGE gels. Alternatively, SEC coupled to MALLS was performed as follows: 100 µl from samples derived from Ni-NTA affinity chromatography was loaded onto a Superdex 200 Increase 10/300 GL SEC prepacked column (GE Healthcare) connected to a high-performance liquid chromatography (HPLC) system (Shimadzu) operating with the LCsolution software and equipped with a solvent delivery module (Shimadzu; LC-20AD), a UV/VIS photodiode array detector (Shimadzu; SPD-M20A) measuring at 280 nm, a differential refractometric detector (Shimadzu; RID-10A), and a system controller (Shimadzu; CBM-20A/20Alite) and coupled to online mass detection by an 8-angle laser light scattering detector (Wyatt; Dawn Heleos 8+). Data were analyzed with the Astra software (ASTRA 6.1.2.84).

### SAXS measurements and modeling.

SAXS data were collected at the SWING beamline of the SOLEIL synchrotron (Gif-sur-Yvette, France) using an Aviex charge-coupled device detector. The measurements were performed at 15°C for three different concentrations of the HrpG/HrpV/HrpJ complex (6.0, 3.0, and 1.5 mg/ml) using the automatic sample changer. The highest-concentration sample was also run through an Agilent HPLC system to assess the behavior of the complex at lower effective concentrations. The sample-to-detector distance was 3.1 m, covering a range of momentum transfer 0.007 < *q* < 0.614 Å^−1^ (*q* = 4*π* sinθ/λ, where 2θ is the scattering angle and λ = 1.033 Å is the X-ray wavelength). Using the Foxtrot software, the data were averaged radially and converted to absolute units, analyzed for radiation damage, averaged, and subtracted. Subsequent analysis was performed with the ATSAS program suite (24). PRIMUS (45) was used for the calculation of the radius of gyration *R*_*g*_ and the forward scattering intensity *I*(0) (proportional to the number of electrons of the particle) from the slope of Guinier plot [ln*I*(*q*) versus *q*^2^] (20). GNOM (22) was used to calculate the pair distance distribution function *p*(*r*) and to estimate the maximum particle dimension (Dmax). The molecular mass (MM) of the solute was estimated from the SAXS data from the *I*(0) (20) and from the hydrated-particle/Porod volume *V* (21), where molecular mass is estimated as *V*/1.6. Homology modeling was conducted through the HHpred server pipeline with MODELLER (46) or with the Sculptor utility (47) of the PHENIX program (48) based on the TyeA/YopN/SycN/YscB complex from Y. pestis (17). The modeling of the missing residues in a way that is compatible with SAXS data was accomplished with CORAL (24).

### MS-based bottom-up proteomic analysis.

The nLC-MS/MS analysis of tryptic peptide mixtures was performed on an Easy-nLC system (Thermo Scientific; software version 2.7.6) coupled with an LTQ-Orbitrap XL ETD (Thermo Scientific, Bremen, Germany) through an nES ion source (Thermo Scientific, Bremen, Germany) as described in reference 49. Samples were reconstituted in 0.5% formic acid aqueous solution, and the tryptic peptide mixtures were separated on a reversed-phase column packed in-house and analyzed by MS as described in references 50 and 51. The nLC-MS/MS raw data were loaded in Proteome Discoverer 1.3.0.339 (Thermo Scientific) and run using the Mascot 2.3.02 (Matrix Science, London, United Kingdom) search algorithm against the *E. amylovora* proteome (last modified 30 November 2016, version 30) containing 8,265 entries and *P. syringae* pv. phaseolicola (strain 1448 A/race 6) proteome (last modified 2 November 2016, version 75) containing 5,046 entries (52). A list of common contaminants was included in the database (53). For protein identification, the following search parameters were used: precursor error tolerance, 10 ppm; fragment ion tolerance, 0.8 Da; trypsin full specificity, maximum number of missed cleavages, 3; and methionine oxidation as variable modifications. Final peptide and protein lists were compiled in Scaffold (version 4.4.1.1; Proteome Software, Portland, OR) employing criteria previously described (49). Protein relative quantitation was performed in Scaffold using different integrated label-free quantitative algorithms.

10.1128/mBio.01096-18.1 TEXT S1  Supplemental materials and methods. Download TEXT S1, PDF file, 0.2 MB. Copyright © 2018 Charova et al. 2018 Charova et al. This content is distributed under the terms of the Creative Commons Attribution 4.0 International license.

10.1128/mBio.01096-18.7 TABLE S1  Strains and plasmids used in this study. Download TABLE S1, PDF file, 0.1 MB. Copyright © 2018 Charova et al. 2018 Charova et al. This content is distributed under the terms of the Creative Commons Attribution 4.0 International license.

10.1128/mBio.01096-18.8 TABLE S2  Primers used in this study. Download TABLE S2, PDF file, 0.1 MB. Copyright © 2018 Charova et al. 2018 Charova et al. This content is distributed under the terms of the Creative Commons Attribution 4.0 International license.
