# Supplementary figures and images for: Migration of Type III Secretion System Transcriptional Regulators Links Gene Expression to Secretion
Source: mBio. 2018 Jul 31;9(4):e01096-18. doi: 10.1128/mBio.01096-18 (PMC6069116; doi:10.1128/mBio.01096-18)

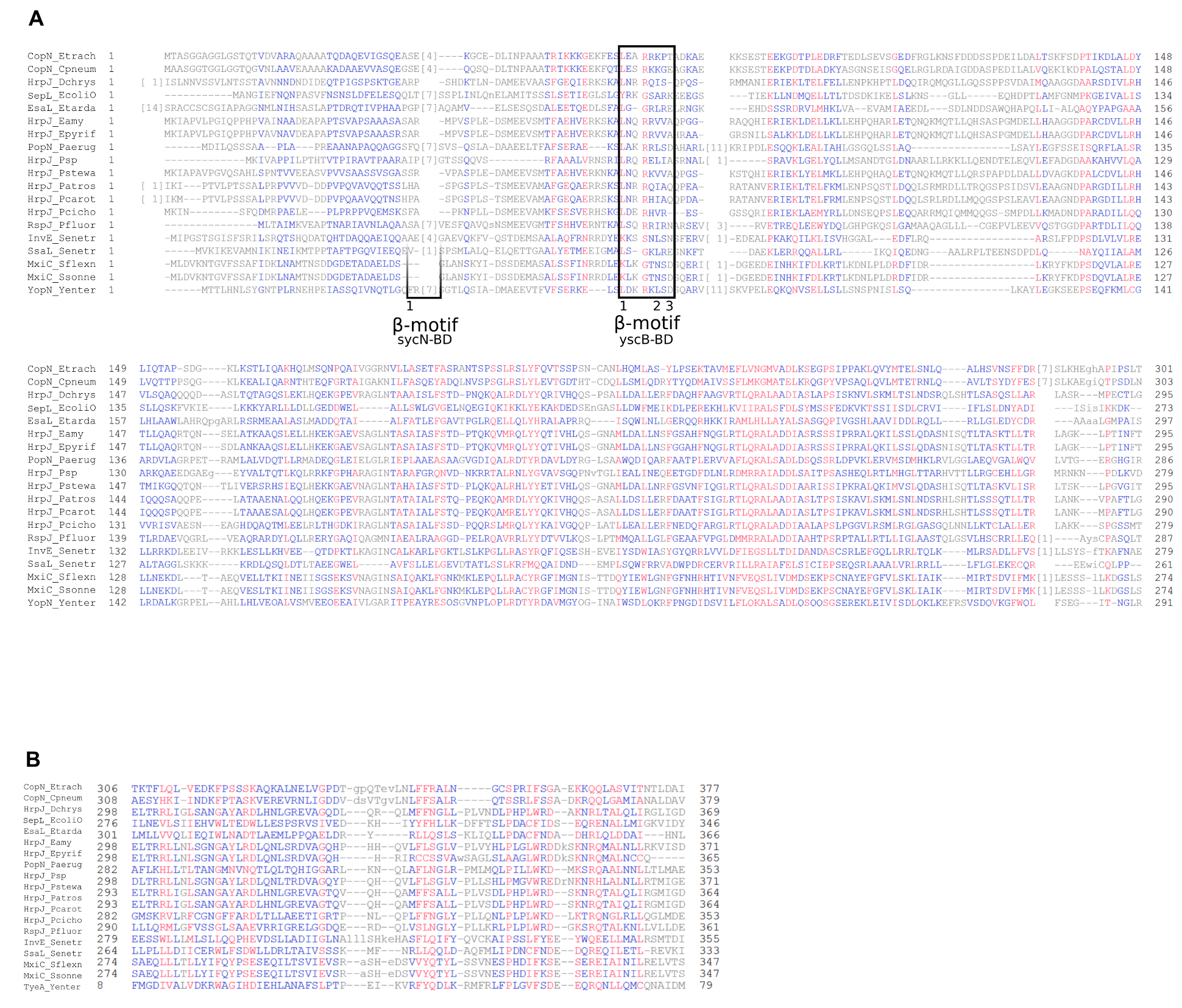

Supplement: FIG S1 [file mbo004184004sf1.tif]

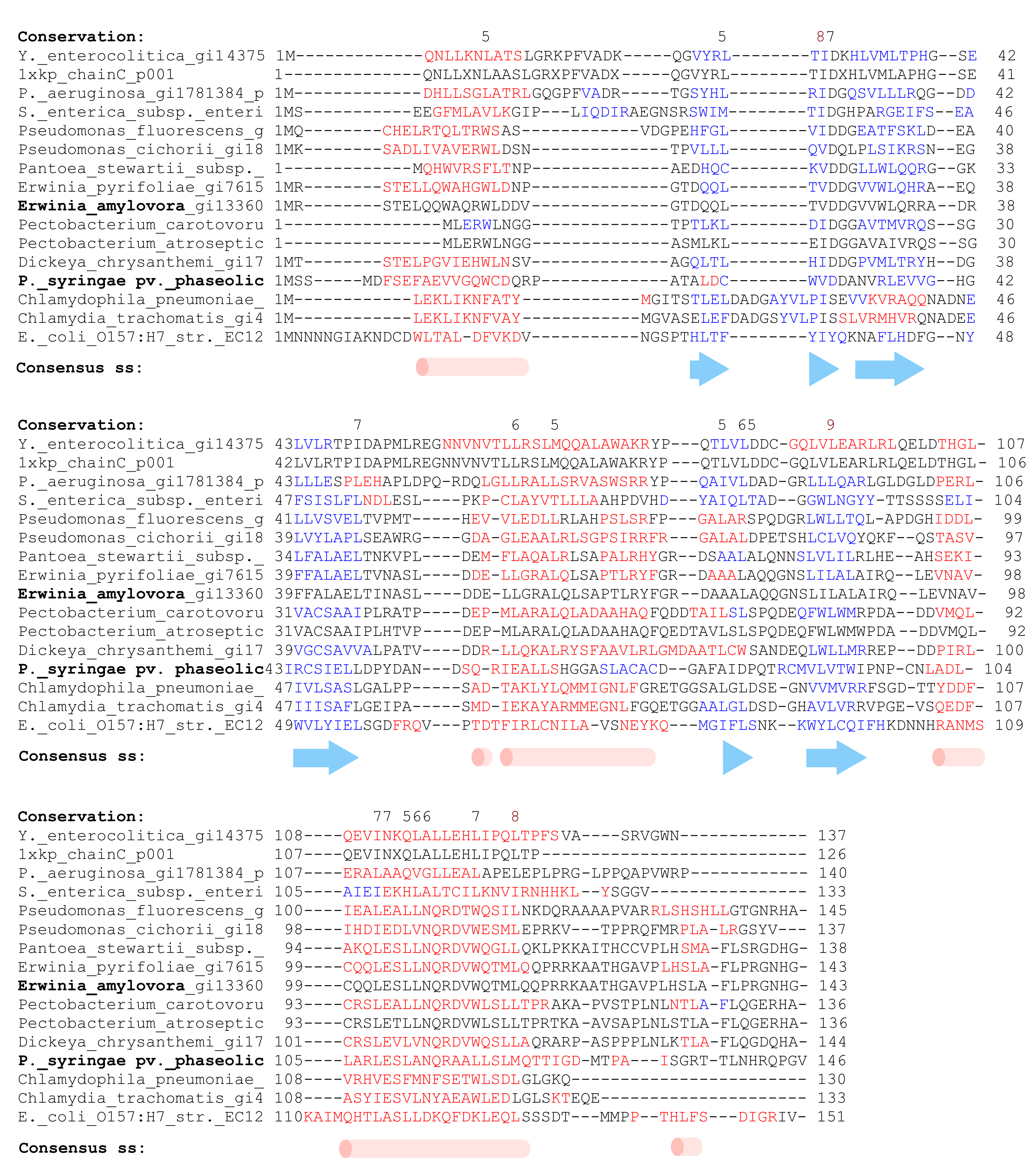

Supplement: FIG S2 [file mbo004184004sf2.tif]

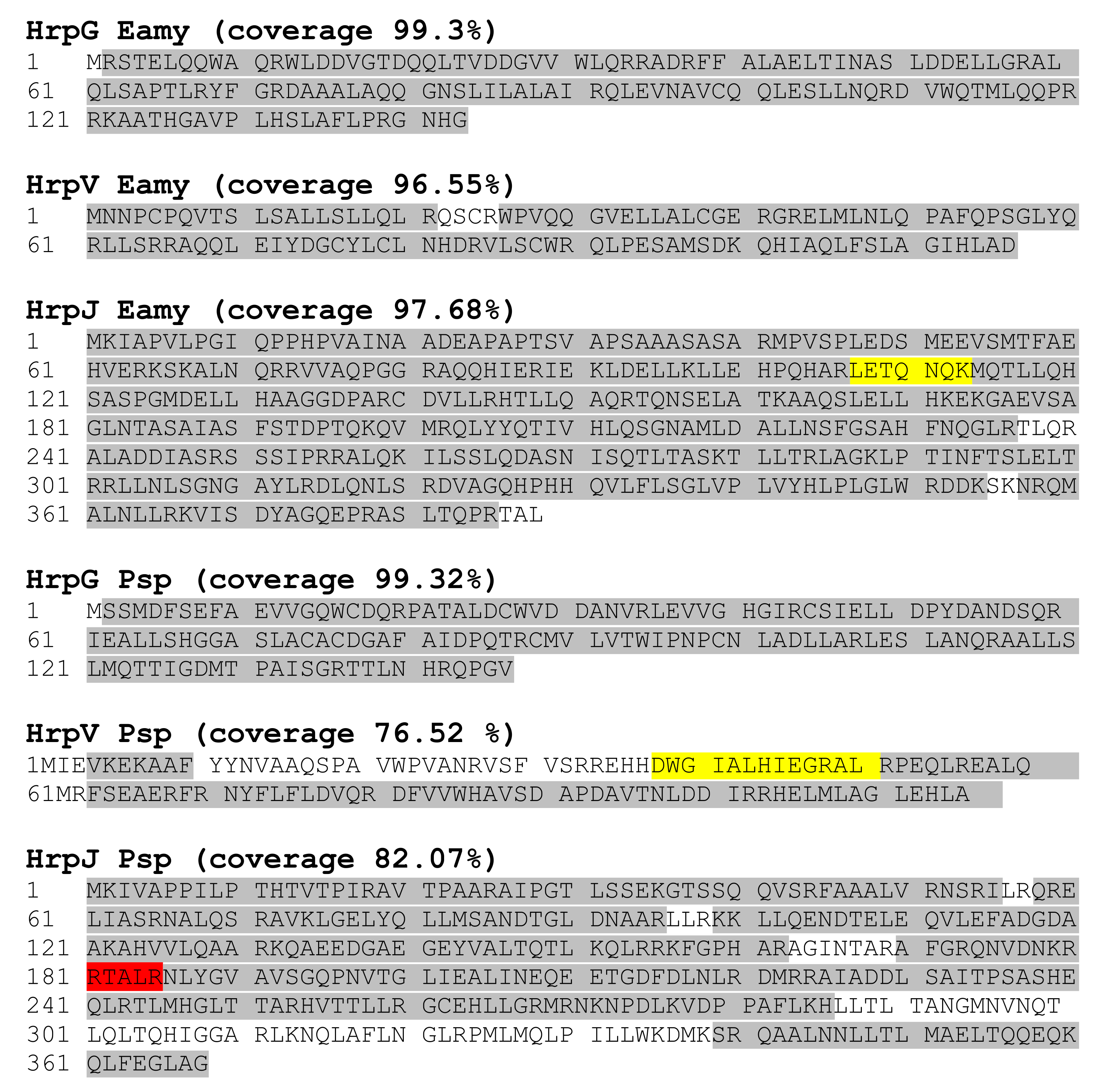

Supplement: FIG S3 [file mbo004184004sf3.tif]

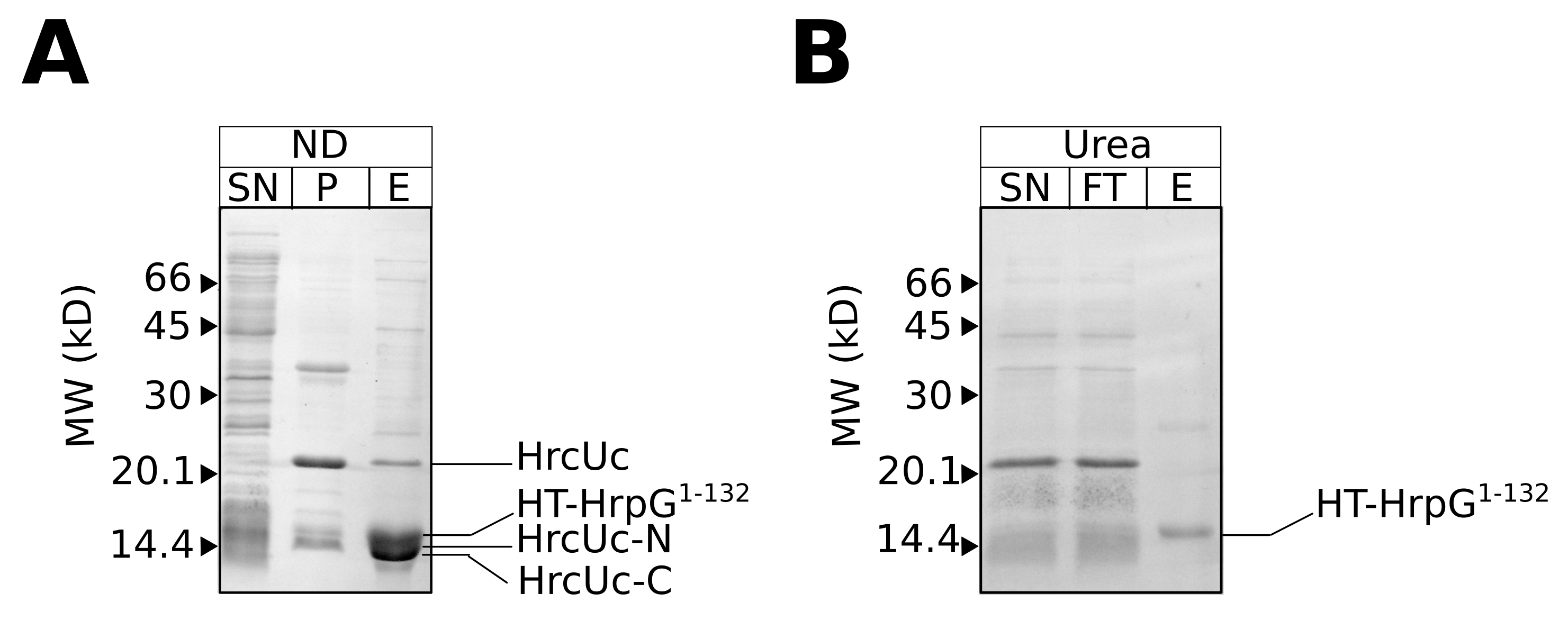

Supplement: FIG S4 [file mbo004184004sf4.tif]

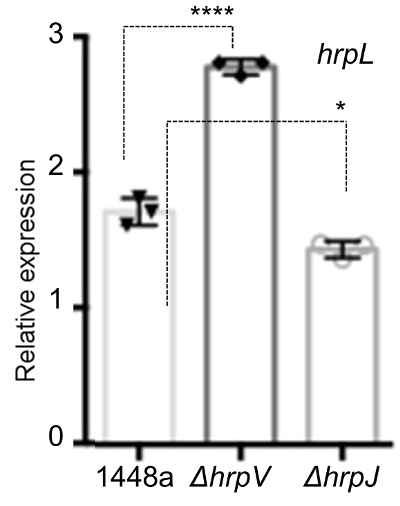

Supplement: FIG S5 [file mbo004184004sf5.tif]
