# Supplementary material for: Migration of Type III Secretion System Transcriptional Regulators Links Gene Expression to Secretion
Source: mBio. 2018 Jul 31;9(4):e01096-18. doi: 10.1128/mBio.01096-18 (PMC6069116; doi:10.1128/mBio.01096-18)
Supplement: TEXT S1 [file mbo004184004s1.pdf]

TEXT S1: Supplemental Materials and Methods

Sequence analysis

Amino acid sequences were retrieved from the National Center for Biotechnology Information and are given in Figures S1 and S2. For class I T3SS chaperones, sequence threading techniques and fold-recognition algorithms were used to identify distant homologs. 3-D structural profiles were predicted from sequence data using the PHYRE pipeline (1). Multiple sequence alignments for class I T3SS chaperones were performed using the PROMALS3D server (2) (Figure S2) to take into account structural information for inferring evolutionary relations. Gatekeeper proteins (SctJ T3SS family of proteins) were aligned either using ClustalW (3) with the COBALT server (4) (Figures S1A and S1B) or using the BLOSSUM matrix (Figure 1B).

The phylogenetic relations were inferred using the Neighbor-Joining method (5). The optimal trees are shown in Figure 1B. The percentage of replicate trees which the associated taxa clustered together in the bootstrap test (1000 replicates) are shown next to the branches (6). The trees are drawn to scale, with branch lengths in the same units as those of the evolutionary distances used to infer the phylogenetic trees. The evolutionary distances were computed using the Poisson correction method (7) and are in units of the number of amino acid substitutions per site. All positions containing gaps and missing data were eliminated. There were a total of 234 positions in the final dataset for gatekeeper proteins and a total of 79 for chaperones. Evolutionary analyses were conducted using the MEGA7 (8) software.

Plasmid construction

The vector used for protein complex production was the bicistronic plasmid pPROpET (9). The *Escherichia coli* strains used for plasmid transformation and subsequently protein expression are documented in S1 Table. Detailed information, on the plasmid sets used in this study, is also provided.

The primers used for subcloning into the two polylinkers of the bicistronic plasmid are shown in S2 Table. With appropriate upper primer modification (inclusion of a ribosome binding site) or usage of pET26b as an intermediate vector, the bicistronic plasmid was transformed to tricistronic, so as to enable the simultaneous production of three polypeptides. Genes were amplified with PCR using as template either pre-existing expression plasmids bearing *Psp* genes or the cosmid pPL6 (10) or *Eamy* genomic DNA isolation. In each resulting expression plasmid, the first protein carries an N-terminal His_6-tag, while the following proteins are untagged. Positive recombinant plasmid clones were verified using restriction enzyme digestion and DNA sequencing.

A 2026 bp tricistron containing *hrpG,hrpV and hrpJ* from *Psp* was designed and optimized for codon usage of *E. coli* host cells (GenScript Biotech). The tricistron was excised from the parental plasmid (pUC57) with enzymes NcoI/EcoRI and cloned to respective sites of pPROPET to generate pPROPET/GVJ_PspGs. From this plasmid, pPROPET/GV_PspGs and pPROPET/GJ_PspGs were generated, after KpnI/EcoRI and XhoI/KpnI excisions respectively, blunt-end generation and re-ligation. For the pET16b/HrpJ_Eamy and pET16b/MBP-TEV/HrpJ_Eamy constructions, HrpJ carrying its native stop codon was excised from pET26b/HrpJ with NdeI/XhoI and ligated to respective sites of pET16b and pET16b/MBP-TEV. $HrpG^{1\text{-}132}$ –was cloned and employed for interaction analysis with the C-terminal domain of HrcU, due to the more favorable solubility properties of the fragment

compared to full length HrpG, to the vector pPROPET/$G^{1\text{-}132}U^{199\text{-}359}$_APTH_Psp. The N265A mutant of $HrcU^{199\text{-}359}$ (HrcUc) was constructed using the QuikChange II Site-Directed Mutagenesis Kit, Agilent Technologies and primers documented in S2 Table.

Native agarose electrophoresis

Using established protocols (11) the separately purified proteins $His_6HrpG^{1\text{-}132}$ and $HrcU^{199\text{-}359}$ were co-incubated for 16 hrs at 4 °C before native agarose gel electrophoresis. Along with the incubated mix, the individual proteins were run side by side as controls.

Western blotting and detection

Gels after electrophoresis were blotted for 1 hour at 4 °C (BIORAD wet transfer system in 1x Tris Glycine buffer in 0.0375% SDS and 20% methanol) on methanol-activated and equilibrated PVDF membranes (Millipore), and the blots for HrpA2 & HrpZ1, were blocked overnight at 4 °C in Tris-buffered saline -Tween20 (0.05% v/v, TBS-T) and 5% non-fat milk. The blots were moved next day, to room temperature and primary polyclonal antibodies were added in a dilution 1:30000 in TBS-T and 5% non-fat milk and incubated for 2-hrs at room temperature. Subsequently, the blots were washed 4 times with TBS-T and secondary antibody was added (anti-rabbit IgG, 1:5000, Sigma) in TBS-T and 5% non-fat milk, followed by an incubation of 1 hr at room temperature. HrpJ blots were blocked for 1 hr at room temperature, followed by an overnight incubation with the primary antibody (1:20000) at 4 °C. Next day the blots were washed as described before and a 3-hrs incubation with the secondary antibody was performed at room temperature (anti-rabbit IgG, 1:5000). Finally, the blots were thoroughly washed and 6 ml of a 1:1 mix of ECL Clarity (BIORAD) developer were added, followed by a 3-min incubation with gentle agitation. Membranes were then transferred to a plastic transparent wrap, air bubbles were removed and signals were monitored and digitally stored using a BIO-RAD gel documentation system (Image Lab™ software, BIO-RAD).

***ΔhrpJ* knockout mutant generation**

To generate the *ΔhrpJ* knockout mutant, an established protocol (12) was followed. Two PCR amplifications were carried out, generating fragments of approximately 500 bp of the 5' and 3' regions flanking *hrpJ*, using *Psp* 1448a genomic DNA as a template. Primers included an EcoRI site and the T7 primer sequence in such a manner so as to provide homology and a cloning site between the fragments: four primers were designed (A1, A2: amplify the 5' region, B1, B2: amplify the 3' region, S1 Table). Primers A2 and B2 share an 18 nts homology. After generation of the A1A2 and B1B2 fragments, the knockout allele was generated using these two fragments as primers/template. The resulting deletion allele was cloned into pGEM-T (Promega) and fully sequenced to discard mutations. An EcoRI-produced fragment containing the flippase recognition target (FRT)-flanked kanamycin resistance gene was obtained from pGEM-T-*nptII*-EcoRI and ligated into the pGEM-T deletion allele digested with EcoRI. Ligated vectors were used to transform *E. coli* DH5α cells and selection was achieved directly on LB-kanamycin plates. Non-polar mutants were verified with Southern analysis and selected for downstream experimentation.

Psp* T3SS induction *in culture

Confluent LB plates of *Psp* 1448a wild-type and *ΔhrpG*, *ΔhrpV*, *ΔhrpJ*, *ΔhrcC* mutants were obtained after overnight incubation. Biomass from the plates was harvested with 10 mM $MgCl_2$, precipitated, washed with 10 mM $MgCl_2$ and resuspended to an optical density of 0.3 in Hrp-inducing medium (HIM) pH 5.8, supplied with 10mM fructose as a carbon source (13). Cultures were incubated at 20 ^{o}C for 6 or 24 hrs with agitation.

RNA extraction and RT-qPCR analysis from *Psp*

10 ml cultures induced for T3SS for 6 hours as described above were used for RNA extraction performed with PURELINK RNA MINI KIT (Life technologies). 1 μg of RNA for each treatment was reverse transcribed using SUPERSCRIPT III 1ST STRAND (Life technologies) according to the manufacturer's instructions.

Quantitative Real-Time PCR (RT-qPCR) was carried out on QuantStudio 12 K FlexReal-Time PCR System (Life Technologies, USA) in 96 well PCR plates, with 25 μL reaction mixture volume, using SYBR SELECT MASTER MIX (Life technologies) according to the manufacturer's instructions. The primers used for Real-Time expression analysis are reported in Table S2. For normalization, the housekeeping gene *16S rDNA* was used. The experiment was performed in triplicate, including standards and negative controls, using three different batches of cDNA obtained from three independent RNA preparations. The PCR protocol was 40 cycles with 95 °C for 20 seconds, 60 °C for 20 seconds, and 72 °C for 20 seconds, after an initial step of 95 °C for 3 min. The amount of fluorescence given by incorporation of the SYBR Green dye into double-stranded DNA was evaluated at the end of each cycle, and analyzed to determine the resulting threshold cycle (Ct) values by Expression Data Analysis Software (Applied Biosystem). Dissociation analysis of amplicons was performed (from 60 to 95 °C, with a 0.5 °C increase every 5 seconds) at the end of each PCR run to check for specific amplifications. The Ct values of each gene tested were normalized to the Ct values of the housekeeping gene, to obtain relative expression data for each gene examined. In order to avoid significant measurement inaccuracies, the amplification efficiency of the primer pair was estimated producing Real-Time PCR curves for a ten-fold dilution series of *Psp* genomic DNA (from 50 ng to 0.5 pg) used as template. The slope of the log-linear phase of each curve reflects the amplification efficiency, which should range between 90% and 100%, with slope value between −3.2 and −3.4, and R2 of at least 0.998. The data obtained represent the mean of three independent replications ± SD.

References

1. Kelley L a, Sternberg MJE. 2009. Protein structure prediction on the Web: a case study using the Phyre server. Nat Protoc 4:363–71.
2. Pei J, Kim BH, Grishin N V. 2008. PROMALS3D: A tool for multiple protein sequence and structure alignments. Nucleic Acids Res 36:2295–2300.
3. Thompson JD, Higgins DG, Gibson TJ. 1994. CLUSTAL W: improving the sensitivity of progressive multiple sequence alignment through sequence weighting, position-specific gap penalties and weight matrix choice. Nucleic Acids Res 22:4673–80.
4. Papadopoulos JS, Agarwala R. 2007. COBALT: Constraint-based alignment tool for multiple protein sequences. Bioinformatics 23:1073–1079.

5. Saitou N, Nei M. 1987. The neighbor-joining method: A new method for reconstructing phylogenetic trees. Mol Biol Evol 4:406–425.
6. Felsenstein J. 2009. Confidence Limits on Phylogenies : An Approach Using the Bootstrap. Society 39:783–791.
7. Zuckerkandl E, Pauling L. 1965. Evolutionary divergence and convergence in proteins, p. 97–166. *In* Vogel, V, Bryson, HJ (eds.), Evolving Genes and Proteins. Academic Press, New York.
8. Kumar S, Stecher G, Tamura K. 2016. MEGA7: Molecular Evolutionary Genetics Analysis version 7.0 for bigger datasets. Mol Biol Evol 33:msw054.
9. Gazi AD, Charova S, Aivaliotis M, Panopoulos NJ, Kokkinidis M. 2015. HrpG and HrpV proteins from the Type III secretion system of *Erwinia amylovora* form a stable heterodimer. FEMS Microbiol Lett 362.
10. Lindgren PB, Peet RC, Panopoulos NJ. 1986. Gene cluster of *Pseudomonas syringae* pv. “phaseolicola” controls pathogenicity of bean plants and hypersensitivity on nonhost plants. J Bacteriol 168:512–522.
11. Kim R, Yokota H, Kim SH. 2000. Electrophoresis of proteins and protein-protein complexes in a native agarose gel. Anal Biochem 282:147–149.
12. Zumaquero A, Macho AP, Rufian JS, Beuzon CR. 2010. Analysis of the role of the type III effector inventory of *Pseudomonas syringae* pv. phaseolicola 1448a in interaction with the plant. J Bacteriol 192:4474–4488.
13. Huynh T V, Dahlbeck D, Staskawicz BJ. 1989. Bacterial blight of soybean: regulation of a pathogen gene determining host cultivar specificity. Science 245:1374–1377.
