# Supplementary material for: Migration of Type III Secretion System Transcriptional Regulators Links Gene Expression to Secretion
Source: mBio. 2018 Jul 31;9(4):e01096-18. doi: 10.1128/mBio.01096-18 (PMC6069116; doi:10.1128/mBio.01096-18)
Supplement: TABLE S1 [file mbo004184004st1.pdf]

S1 Table. Strains and plasmids used in this study

Strains	Description	Reference
Escherichia coli		
DH5a	F^- *endA1 glnV44 thi-1 recA1 relA1 gyrA96 deoR nupG purB20* φ80d*lacZ*ΔM15 Δ(*lacZYA-argF*)U169, hsdR17($r_K^- m_K^+$), λ^-	(1)
BL21(DE3)	F^- *ompT gal dcm lon hsdS*$_B$($r_B^- m_B^-$) λ(DE3 [*lacI lacUV5-T7p07 ind1 sam7 nin5*]) [*malB*$^+$]$_{K\text{-}12}$(λ^S)	(2)
Pseudomonas syringae pv. phaseolicola		
1448a	Race 6, wild type strain	(3)
IOM71	*ΔhrpG,* race 6 derivative (Km^R)	(4)
IOM48	*ΔhrpV,* race 6 derivative (Km^R)	(4)
Pph *ΔhrpJ*	*ΔhrpJ,* race 6 derivative (Km^R)	This work
Pph *ΔhrcC*	*ΔhrcC,* race 6 derivative (Km^R)	(5)
Erwinia amylovora		
ATCC 49946	Strain ATCC 49946, wild type	(6)
**Plasmids**	**Description**	**Reference**
pPROEX-HTb	Cloning vector, Amp^R	Invitrogen
pET16b	Cloning vector, Amp^R	Novagen
pET26b	Cloning vector, Km^R	Novagen
pET16b/MBP-TEV	Cloning vector, Amp^R	Prof. A. Economou
pPROPET	Cloning vector, Km^R	(7)
pBBR1-MCS-4	Cloning vector, Amp^R	(8)
pPL6	Cosmid containing the *hrp*/*hrc* pathogenicity island from *Psp3121,* Trc^R	(9)
pET16b/HrpJ_Eamy	pET16b expressing His_{10}HrpJ from *Eamy*, Amp^R	This work
pET16b/MBP-TEV/HrpJ_Eamy	pET16b expressing His_{10}MBP-TEV-HrpJ fusion from *Eamy*, Amp^R	This work
pPROPET/GVJ_PspGs	pPROPET co-expressing His_6HrpG,HrpV,HrpJ from *Psp* as a tricistronic plasmid-genes optimized for codon usage (Genescript), Km^R	This work
pPROPET/GV_PspGs	pPROPET co-expressing His_6HrpG,HrpVfrom *Psp* as a bicistronic plasmid-genes optimized for codon usage (Genescript), Km^R	This work
pPROPET/GJ_Psp	pPROPET co-expressing His_6HrpG,HrpJ from *Psp* as a bicistronic plasmid, Km^R	This work
pPROPET/VJ_Psp	pPROPET co-expressing His_6HrpV,HrpJ from *Psp* as a bicistronic plasmid, Km^R	This work
pPROPET/GVJ_Eamy	pPROPET co-expressing His_6HrpG,HrpV,HrpJ from *Eamy* as a tricistronic plasmid, Km^R	This work
pPROPET/GV_Eamy	pPROPET co-expressing His_6HrpG,HrpVfrom *Eamy* as a bicistronic plasmid, Km^R	This work
pPROPET/GJ_Eamy	pPROPET co-expressing His_6HrpG,HrpJ from *Eamy* as a bicistronic plasmid, Km^R	This work
pPROPET/VJ_Eamy	pPROPET co-expressing His_6HrpV,HrpJ from *Eamy* as a bicistronic plasmid, Km^R	This work
pPROPET/$G^{1\text{-}132}U^{199\text{-}359}$_Psp	pPROPET co-expressing His_6HrpG $^{1\text{-}132}$, $HrcU^{199\text{-}359}$ from *Psp*, Km^R	This work
pPROPET/$G^{1\text{-}132}U^{199\text{-}359}$_APTH_Psp	pPROPET co-expressing His_6HrpG$^{1\text{-}132}$, $HrcU^{199\text{-}359}$ the latter carrying a N265A mutation, from *Psp*, Km^R	This work
pPROPET/$GU^{199\text{-}360}$_Eamy	pPROPET co-expressing His_6HrpG, $HrcU^{199\text{-}360}$ from *Eamy*, Km^R	This work

pET16b/HrcU$^{199-359}$_Psp	pET16b expressing $His_{10}HrcU^{199-359}$ from *Psp*, Amp^R	Prof. A. Tampakaki
pET16b/HrpA2_Psp	pET16b expressing His_{10}HrpA2 from *Psp*, Amp^R	Prof. A. Tampakaki
pET26b/HrpZ1_Psp	pET26b expressing HrpZ1His_6 from *Psp*, Km^R	Prof. A. Tampakaki
pET26b/HrpJ_Eamy	Intermediate plasmid, donor of HrpJ insert for pPROPET/GVJ_*Eamy* plasmid construction, Km^R	This work

References

1. Grant SGN, Jessee J, Bloom FR, Hanahan D. 1990. Differential plasmid rescue from transgenic mouse DNAs into Escherichia coli methylation-restriction mutants. Proc Natl Acad Sci U S A 87:4645–4649.
2. Wood WB. 1966. Host specificity of DNA produced by Escherichia coli: bacterial mutations affecting the restriction and modification of DNA. J Mol Biol 16:118–133.
3. Joardar V, Joardar V, Lindeberg M, Lindeberg M, Jackson R, Jackson RW, Selengut J, Dodson R, Brinkac LM, Daugherty SC, Deboy R, Durkin a S, Giglio MG, Madupu R, Nelson WC, Rosovitz MJ, Sullivan S, Crabtree J, Creasy T, Davidsen T, Haft DH, Zafar N, Zhou L, Halpin R, Holley T, Khouri H, Feldblyum T, White O, Fraser CM, Chatterjee AK, Cartinhour S, Schneider DJ, Collmer A, Buell CR. 2005. Whole-Genome Sequence Analysis of. J Bacteriol 187:6488–6498.
4. Ortiz-Martin I, Thwaites R, Mansfield JW, Beuzon CR. 2010. Negative regulation of the Hrp type III secretion system in Pseudomonas syringae pv. phaseolicola. Mol Plant Microbe Interact 23:682–701.
5. Ortiz-Martín I, Macho AP, Lambersten L, Ramos C, Beuzón CR. 2006. Suicide vectors for antibiotic marker exchange and rapid generation of multiple knockout mutants by allelic exchange in Gram-negative bacteria. J Microbiol Methods 67:395–407.
6. Sebaihia M, Bocsanczy AM, Biehl BS, Quail MA, Perna NT, Glasner JD, DeClerck GA, Cartinhour S, Schneider DJ, Bentley SD, Parkhill J, Beer S V. 2010. Complete genome sequence of the plant pathogen Erwinia amylovora strain ATCC 49946. J Bacteriol 192:2020–2021.
7. Gazi AD, Charova S, Aivaliotis M, Panopoulos NJ, Kokkinidis M. 2015. HrpG and HrpV proteins from the Type III secretion system of Erwinia amylovora form a stable heterodimer. FEMS Microbiol Lett 362.
8. Kovach ME, Elzer PH, Hill DS, Robertson GT, Farris MA, Roop RM, Peterson KM. 1995. Four new derivatives of the broad host range cloning vector PBBR1MCS, carrying different antibiotic resistance cassettes. Gene 166:175–176.
9. Lindgren PB, Peet RC, Panopoulos NJ. 1986. Gene cluster of Pseudomonas syringae pv. “phaseolicola” controls pathogenicity of bean plants and hypersensitivity on nonhost plants. J Bacteriol 168:512–522.
