# Supplementary material for: Migration of Type III Secretion System Transcriptional Regulators Links Gene Expression to Secretion
Source: mBio. 2018 Jul 31;9(4):e01096-18. doi: 10.1128/mBio.01096-18 (PMC6069116; doi:10.1128/mBio.01096-18)
Supplement: TABLE S2 [file mbo004184004st2.pdf]

S2 Table. Primers used in this study

pPROPET co-expression plasmids	MCS1	MCS2
pPROPET/G$^{1\text{-}132}$U$^{199\text{-}359}$_Psp	*hrpG*$^{1\text{-}132}$ (template pPROEX/*hrpG*)<br><br>Forward: 5’ – GACCAATCATGAGCTCGATGGATTTC–3’ (ligated to compatible vector’ s NcoI site)<br><br>Reverse: 5’ – AGGGTCGTGCGGCCGCATCACGCGG- 3’	*hrcU*$^{199\text{-}359}$ (template pET16b/*hrcU*$^{199\text{-}359}$)<br><br>Forward: 5’ - CTGGTGCAAGTCATATGAGTGAAAAAACCGA-3'<br><br>Reverse: 5’-CCTGTTACGCCTGGATCCCAGCTCC-3’
pPROPET/VJ_Psp	*hrpV* (template pET16b/*hrpV*)<br><br>Forward: 5’ – AGGATTAGTCATGATTGAGGTAAAG–3’ (ligated to compatible vector’ s NcoI site)<br><br>Reverse: 5’ – CGTCTAGAAGCTTGCGTCC–3’	*hrpJ*<br>(template pPROEX/*hrpJ*)<br><br>Forward: 5’–GCTCAGAACCGTCATATGAAAATCG–3’<br><br>Reverse: 5’–CGACAGGGCAAGCTTGTTCAGAAAG–3’ (ligated blunt to vector’ s SacI site after Klenow treatment)
pPROPET/GJ_Eamy	*hrpG* (template: genomic DNA *Eamy*)<br><br>Forward: 5’ – GCCATCATTCATGAGATCAACTGAATTGCAGC – 3’<br><br>Reverse: 5’ - GCAACTCTCGAGTCTCAACCATGGTTTCC -3’	*hrpJ* (template: genomic DNA *Eamy*)<br><br>Forward: 5’ – GAAGGTTCGCCCATATGAAAATTGC – 3’<br><br>Reverse: 5’ - GCCAGACAAACAGAGCTCTCATTTA - 3’
pPROPET/GV_Eamy	*hrpG*<br>(as in 3)	*hrpV* (template: genomic DNA *Eamy*)<br><br>Forward: 5’-GTATCGGCATATGAATAATCCTTGC-3’<br><br>Reverse: 5'-CCGGC GAATTCAGAAAAAATAACC-3'
pPROPET/GVJ_Eamy	*hrpG*<br>(as in 3)	*hrpV/hrpJ*<br>*hrpV* as in 4.<br>*hrpJ* was first subcloned to pET26b using primers in 3, digested from there XbaI(blunt)/SacI and ligated to pPROpET/*hrpG*/*hrpV* in sites EcoRI(blunt)/SacI)
pPROPET/VJ_Eamy	*hrpV*(template genomic DNA *Eamy*)<br><br>Forward: 5’ – GGTATCGGTCCATGGATAATCCTTG – 3’<br><br>Reverse: 5’ - GAAAGGACGCAAGCTTGGATCAATC -3’	*hrpJ* (template genomic DNA *Eamy*)<br>(as in 3)
pPROPET/GU$^{199\text{-}360}$_Eamy	*hrpG*<br>(as in 3)	*hrcU*$^{199\text{-}360}$ (template: genomic DNA *Eamy*)<br><br>Forward: 5’ - GCTGGTACTGCATATGCTGGACTTCGGCC -3'<br><br>Reverse: 5’- GGTAATAGTTGAGCTCATTCGGCTTCCAGTTC -3’

**Site directed mutagenesis plasmid 1 derivative**	**Forward primer**	**Reverse primer**
pPROPET/G$^{1-132}$U$^{199-359}$_APTH_Psp	5’ – GACATGCTGCTGGTGGCGCCGACGCACTAC GCGG- 3’	5’- CCGCGTAGTGCGTCGGCGCCACCAGCAGCATGTC-3’
***Psp hrpJ* knock-out allele generation**		
A1	5’-CTGGCGATACATTTTGCGG-3’	
A2	5’-CCCTATAGTGAGTCGAATTCAGGACGGTTCTGAGCCTG-3’	
B1	5’-CAGGTTGGCGCGCAGATC-3’	
B2	5’-GAATTCGACTCACTATAGGGATCAAGTCATCAACTTTC-3’	
**RT-qPCR analysis**	**Forward primer**	**Reverse primer**
hrpL (159 bp amplicon)	5’ –AGCCGCAGACCTGGTTGTG- 3’	5’ -ATTGCCTGTGCCCGTCTACC3-’
16S rDNA *(157 bp amplicon)*	5’-GGAATCTGCCTGGTAGTGGGG- 3’	5’-GGCTCACCAAGGCGACGAT 3-’
